# Colonization and Interaction of Bacteria Associated With Chinese Chives Affected by Ecological Compartments and Growth Conditions

**DOI:** 10.3389/fmicb.2022.775002

**Published:** 2022-02-14

**Authors:** Nan Sun, Yuxin Wang, Jianhua Chen, Pingzhi Wang, Weitang Song, Peifang Ma, Yabin Duan, Ziyuan Jiao, Yixiao Li

**Affiliations:** ^1^College of Water Resources and Civil Engineering, China Agricultural University, Beijing, China; ^2^Pingdingshan Academy of Agricultural Sciences, Pingdingshan, China

**Keywords:** Chinese chive, endophyte, ecological compartment, growth condition, colonization, interaction

## Abstract

Chinese chive has a long history of planting in China. At present, there are many studies on endophytic bacteria and rhizosphere microorganisms of Chinese chive, but the effects of ecological compartment and growth conditions on bacterial communities in Chinese chives are unclear. Here, we aimed to elucidate the differences in bacterial a-diversity, β-diversity, community structure, core species differences, interaction networks and predicted metabolic functions among bacterial communities in different ecological compartments (the phylloplane, leaf endosphere, stem endosphere, root endosphere, and rhizosphere) in Chinese chives in an open field, a solar greenhouse, an arched shed, and a hydroponic system. Sixty samples were collected from these five ecological compartments under four growth conditions, and we compared the bacterial profiles of these groups using 16S rRNA sequencing. We evaluated the differences in diversity and composition among bacterial communities in these ecological compartments, analyzed the bacterial interaction patterns under the different growth conditions, and predicted the bacterial metabolic pathways in these ecological compartments and growth conditions. The results showed that the effects of ecological compartments on bacterial diversity, community composition, interaction network pattern, and functional expression of Chinese chives were greater than those of growth condition. Ecological compartments (*R*^2^ = 0.5292) could better explain bacterial community division than growth conditions (*R*^2^ = 0.1056). The microbial interaction networks and indicator bacteria in different ecological compartments showed that most of the bacteria that played the role of key nodes (OTUs) in each ecological compartment were bacteria with high relative abundance in the compartment. However, the bacteria that played the role of key nodes (OTUs) in bulbs were not Proteobacteria with the highest relative abundance in the compartment, but Actinobacteria that were significantly enriched in the root endosphere and rhizosphere ecological compartments. In addition, interactions among bacteria were interrupted in the hydroponic system, and specific bacterial communities and interaction patterns in Chinese chives varied among growth conditions. Prediction of metabolic functions indicated that plant metabolic activity related to stress responses and induction of system resistance was greater in belowground ecological compartments.

## 1. Introduction

Chinese chives (*Allium tuberosum* Rottl. ex Spreng.) is a perennial plant species in which the leaves are flat and long, succulent and pungent, and the flowers are mostly white. Chinese chives are rich in nutrients, containing iron, zinc, and other trace elements (Wang et al., 2017a), and high in calcium content, which can effectively prevent iron deficiency anaemia. Glutamic acid, proline, and alanine are abundant in Chinese chive leaves (J.Y. et al., 2021), so this plant is a high-quality amino acid provider. The leaves of Chinese chives also contain high levels of total dietary fiber and insoluble dietary fiber, which can effectively promote gastrointestinal peristalsis (Dai and Chau, 2017) and thus reduce the risk of colorectal cancer (Wu et al., 2019). In recent years, the medicinal value of Chinese chives has attracted increased attention from scholars in various countries. The content of unsaturated fatty acids in Chinese chive seeds is approximately 85–90%, mainly in the form of the polyunsaturated fatty acid linoleic acid (Sun et al., 2014a), which can reduce levels of cholesterol and triglycerides in the human body and help regulate the concentration of blood lipids after eating (Wang et al., 2014). Chinese chives have long been cultivated in China and are the most widely distributed among all vegetables, and even on the Qinghai-Tibet Plateau, considerable area is planted in Chinese chives. Therefore, research on the diversity of endophytic bacteria in this plant is highly applicable to agricultural practice.

Endophytes are microorganisms (mainly fungi and bacteria) that live inside healthy plants during almost all life cycle stages without harming the host plants (Yang and Cao, 2016). Endophytes are present in numerous tissues and organs in plants and constitute a natural component of plants. In recent years, endophytes (Jagannath et al., 2019) isolated from bulbs of Liliaceae have been found to synthesize abundant growth promotion hormones (Khan et al., 2020), such as indole acetic acid, that can promote plant growth (Lang, 2018). A highly significant difference in chlorophyll content has been found in maize infected by endophytic bacteria, which indicates that endophytic bacteria can effectively (Emami et al., 2020) stimulate the synthesis of chlorophyll in plants, thereby promoting photosynthesis and growth. Endophytic fungi and bacteria also increase phosphorus efficiency and the N-use efficiency index (Da Silveira et al., 2019) in host plants, and some endophytic bacteria planted on the leaves of host plants can inhibit the infection and growth of pathogens in plants by secreting specific metabolites to form competitive advantages (Moin et al., 2020; Chaouachi et al., 2021). Therefore, microbial endophytes should be further studied for their potential in plant control disease.

Previous studies have found that intercropping cabbage and Chinese chives crops can enhance their quality by increasing the content of vitamin c and soluble sugar in cabbage (Ping, 2020), and reduce the incidence of diseases (Xu et al., 2016) such as cabbage soft rot (Li et al., 2020). At the same time, Chinese chives intercropping with other crops can effectively improve the activity of urease and phosphatase in soil (Meng ZiLi, 2018). Researchers have speculated that Chinese chives can also inhibit the occurrence of soft rot. Further studies have shown that extracts of Chinese chives plants can significantly reduce decay from bacterial soft rot resulting from infection by *Erwinia carotovora* (Ndivo et al., 2018) or *Pectobacterium carotovorum* (Simeon and Abubakar, 2014). Moreover, rotation of Chinese chives with other crops affects crop soil microbial quantity, species, community structure, and diversity (Gu et al., 2020). Therefore, it is speculated that Chinese chives contain endophytic bacteria or associate with rhizospheric microorganisms that can inhibit pathogens. In the microbial analysis of various ecological compartments of other plants, it can be found that ecological compartments have effects on microbial composition and microbial community function (Wei et al., 2021). However, the current studies on plant ecological compartments are mostly between underground compartments (especially in the rhizosphere and root surface), and the composition differences of soil microorganisms, rhizosphere microorganisms, and root endosphere microorganisms and the screening mechanism of roots are studied (Reinhold Hurek et al., 2015). At present, the articles on the effects of ecological compartments on plant microorganisms mainly focus on one of the studies on the differences in microbial community composition, interaction network, and function among ecological compartments. There are few articles that comprehensively analyze the differences among ecological compartments of plants. Similar to the decrease of microbial diversity from rhizosphere to root endosphere (Edwards et al., 2015) and significant differences in microbial composition in underground compartments, we believe that different ecological compartments have effects on bacterial community composition and function of Chinese chives. Previous studies have shown that different facilities will affect the soluble sugar content, vitamin c content and propylene glycol content of plants (Sun et al., 2014b; Chao and Yingcui, 2019), thereby affecting the quality. In our cultivation base, due to the lack of heat preservation wall on the north side, the air temperature and relative humidity in the arched shed are quite different from those in the solar greenhouse (Supplementary Table S1). Therefore, we believe that the large-scale growth condition compared with the small-scale ecological compartment also affects the community distribution and interaction of phylloplane, rhizosphere, and endophytic bacteria in Chinese chives.

In this study, the 16S rRNA V5-V6 gene sequences of Chinese chives bacteria were sequenced using Illumina-MiSeq to compare the differences in bacterial diversity, community composition, interaction network pattern, and functional expression of phylloplane, rhizosphere, and endophytic bacteria of Chinese chives in different ecological compartments and growth conditions. Filling the current research gap on endophytes in Chinese chives is helpful for us to analyze the abundance changes of differential microbiota at the level of bacterial distribution, and provide a theoretical basis for the subsequent isolation of endophytes in Chinese chives.

## 2. Methods

### 2.1. Sample Plots and Methods

Sampling was conducted in an open field, a solar greenhouse, an arched shed (Supplementary Table S1) and a hydroponic system under similar production management at Lizhuang Planting Base (33°66′N, 113°26′E) of Pingdingshan Academy of Agricultural Sciences, Pingdingshan, Henan Province, China, and the area planted in Chinese chives in the solar greenhouse, arched shed and field exceeded 600 m^2^. The Chinese chives in the field was planted in spring, and the Chinese chives in greenhouse and arch shed was planted in winter. All Chinese chives in the three cultivation environments were cultivated in soil. Fertilization management: a compound water-soluble fertilizer (N: P2O5:K2O=20%: 20%: 20%, 375–450 kg/ha) was used as the base fertilizer, applied once in spring and twice in autumn. Nutrient components in hydroponics: Ca(NO3)2.4(H2O) 425 mg/kg, KNO3 668 mg/kg, KH2PO4 200 mg/kg, (NH4)2SO4 380 mg/kg, K2SO4 116 mg/kg, MgSO4 185 mg/kg. In December 2019, a total of 60 samples (5^*^4^*^3) were collected in each growth condition, including 3 duplicate plant samples (complete Chinese chives containing leaves, roots, and stems), 3 rhizosphere soil samples (3 water samples near the roots were collected in a sterile test tube in a hydroponic environment) and 3 phylloplane microbial samples (wiped with sterile cotton swabs). Sampling of plants followed this protocol: healthy Chinese chives about 1 year of age and with growth consistent with surrounding Chinese chives were selected from each growth condition, which were planted homogeneously in Chinese chives, and the sampling distances within each growth condition exceeded 100 m. The rhizospheric soil sampling followed the following (Courchesne and Gobran, 1997) method: healthy roots were excavated along the base of each sampled plant, loose soil attached to the root was gently shaken off, leaving the soil closely bound to the root (about 1 mm attached to the root), then the root samples were sealed in sterile plastic bags, samples were transferred to a car refrigerator and transported to the laboratory, and the rhizospheric samples from the hydroponic environment were obtained by absorbing water samples near the roots (Chi et al., 2012).

### 2.2. Study Subjects and Biopsy Collection

In this study, rhizospheric soil samples from the same growth condition were mixed evenly, then the animal and plant residues visible in the soil were removed, and finally combined samples were screened with a 0.2-mm sieve. The collected Chinese chive samples were rinsed with tap water and then rinsed in distilled water three times (Zhu et al., 2020). This was done to fully remove materials attached to leaves, bulbs and roots. Then, clean Chinese chive leaves, bulbs, and roots were placed in 50-mL Falcon centrifuge tubes each containing 25 mL PBS buffer, shaken at 180 r/min for 15 min, and cleaned a total of three times. Samples were then dried by sterile filter paper, and impurities in the samples were removed by sterile tweezers. Finally, Chinese chive leaves, bulbs, roots, and corresponding soil were stored at −80°C. These samples from different ecological compartments were used to extract plant and soil microbial genomic DNA, and high-throughput sequencing (HTS) was performed.

### 2.3. DNA Extraction

First, the frozen samples were homogenized with an MP Fastprep-24 5G (MP Biomedicals, USA) at 7200 r/min 4 times for 30 s each time. Then, according to the manufacturer's instructions, the DNA of bacteria in Chinese chive leaves, bulbs, and roots, soil and water samples was extracted by the FastDNA®SPIN Kit for Soil (MP Biomedicals, USA). Finally, the quality and concentration of DNA were determined by NanoDrop 2000 UV-Vis spectrophotometry (Thermo Scientific, USA), and DNA fragments were separated by 1% agarose gel electrophoresis. High-quality DNA templates were diluted to 10 ng/μl for subsequent PCR of 16S rRNA.

### 2.4. Bacterial 16s rRNA Amplification and Sequencing

Nested PCR (Xia et al., 2018) was used in this study. Degenerate PCR primers 799F-1392R with a barcode added at the 5′ ends to distinguish different samples were used in the first round. Each PCR amplification system (20 μL) contained 5 × Fast Pfu Buffer (4μL), 2.5 mmol/L dNTPs (2 μL 5 μmol/L forward primer (0.8 μL), 5 μmol/L reverse primer (0.8 μL), FastPfu DNA Polymerase (0.4 μL), BSA (0.2 μL), template DNA (10 ng), and sufficient ddH2O to bring the volume to 20 μL. The PCR amplification procedure was as follows: predenaturation at 95°C for 3 min; 27 cycles of denaturation at 95°C for 30 s, annealing at 55°C for 30 s, and extension at 72°C for 45 s); followed by a final extension at 72°C for 10 min in the analyzer (ABI GeneAmp® 9700, USA). For the second round of PCR, primers were 799F-1193R with a barcode added at the 5′ ends to distinguish different samples for amplifying the V5-V7 hypervariable regions of the bacterial 16S rRNA gene (Wang et al., 2020c), and the total volume of each PCR amplification system was also 20 μL. The PCR amplification procedure in the second round was as follows: predenaturation at 95°C for 3 min; 13 cycles of denaturation at 95°C for 30 s, annealing at 55°C for 30 s, and extension at 72°C for 45 s; followed by final extension at 72°C for 10 min.

Three PCR amplifications were performed per sample, and the PCR products were detected by 2% agarose gel electrophoresis. The target length segment (~400 bp) was cut from each gel with an AxyPrep DNA Gel Extraction Kit (Axygen Biosciences, USA), eluted with Tris-HCl, and detected by 2% agarose gel electrophoresis. Then, the PCR products were detected and quantified by a Quantus.Fluorometer (Promega, USA). Finally, a library was constructed with the NEXTflex® Rapid DNA-Seq Kit, and the purified PCR products were sequenced (2 × 300 nt) on the Illumina MiSeq platform (Illumina, San Diego, USA).

### 2.5. Bacterial 16s rRNA Sequence Analysis

After constructing an HTS reference dataset, data were analyzed by Wang et al. (2018) fastp software and FLASH software (http://www.cbcb.umd.edu/software/flash, version 1.2.7). The process is described below. First, the barcode was removed from the sequences, and quality control and splicing of sequencing sequences at both ends were performed. Each sample was distinguished according to the barcode sequence, and then the chimera was identified and removed to obtain the optimized sequence. Chimera recognition was based on the SILVA database, and then with this database as the reference sequence, the nucleotide sequences with lengths greater than 250 bp were classified into operational taxonomic units (OTUs) with 97% similarity by UPARSE software (http://drive5.com/uparse/) and used for analysis of relative abundances of bacterial taxa in different growth condition samples.

Finally, the sequence data were compared with the Silva (Release.138) 16S rRNA gene database. The algorithm applied was Rdp Classifier (http://rdp.cme.msu.edu), and the confidence threshold was set to 70% (Amato et al., 2013) to determine the taxonomic status of microorganisms corresponding to sequences.

### 2.6. Statistical Analysis

The Kruskal-Wallis H test and Tukeys HSD test were used to evaluate differences in alpha index values (Tan et al., 2021), and bar charts were used to show the relative abundances of phyla and genera in different ecological compartments and different growth conditions. The top 50 bacterial OTUs were selected for each ecological compartment, and IQ-Tree software was utilized to construct phylogenetic trees (Katoh et al., 2002; Price, 2009). Then, the Interactive Tree of Life (ITOL) (Letunic and Bork, 2007) website was used for visualization. Based on the Bray-Curtis method, a principal coordinate analysis (PCOA) (Lozupone and Knight, 2005) map was drawn by using the relative abundances of OTUs in different ecological compartments and growth conditions.

Based on OTUs, a Venn diagram was drawn for analysis of group-specific bacterial microbiota. Bubble diagrams were used to show indicator species in each ecological compartment. The abundance of the bacteria at the genus level was analyzed by using QIIME2 and the vegan package in R software. The Kruskal-Wallis test and Wilcoxon rank sum test (between two groups) were used to evaluate the intergroup significant differences at the phylum and genus levels based on quadrat community abundance data. P values were corrected using the false discovery rate (FDR) (Liu et al., 2007), and Tukey-Kramer was used for multiple comparisons. Average values of samples within groups were calculated, analyzed, and visualized by STAMP and R software (R 4.0.3 statistical package).

To examine the microbial interaction patterns under different growth conditions, the Spearman method was used to analyze correlations between bacterial abundances at the genus level. Coefficients of correlation between bacterial abundances were calculated by the psych package of R. Cytoscape 3.8.2 was released to visualize the patterns of microbial interaction networks, showing only significant correlations (*P* < 0.05). MENA was used to analyze the microbial interaction network between different ecological compartments, and the OTU of more than 6 times in 12 samples was analyzed. Randomize the network structure and then calculate network properties in MENA. Visual analysis was performed by Gephi 0.9.2. Within-module connectivity (Zi) and between-module connectivity (Pi) are used for key nodes (OTUs) analysis.

Tax4Fun software was used to predict gene functions from 16s rRNA data based on the Silva database to obtain the metabolic pathway composition of microorganisms in each ecological compartment and growth condition, and Statistical Analysis of Metagenomic Profiles (STAMP 2.1.3) and SPSS Statistics (SPSS 25) were used for statistical analysis and mapping. STAMP was used for pairwise comparisons of ecological compartments and growth conditions, and Tukey-Kramer was used to adjust for multiple comparisons. The effect size for differences in proportions between groups was greater than 0.05.

## 3. Results

### 3.1. Sequencing Results for Microorganisms in Chinese Chives

A total of 3, 419, 941 reads with an average length of 377 bp were obtained by 16S rRNA high-throughput sequencing analysis of bacterial communities from 60 samples of Chinese chive plants and rhizospheres. Among them, a total of 2, 499, 596 effective sequences (effective rate 73.09%) were obtained, and the original sequences were then grouped and filtered by barcode tag sequence (Table 1). When samples were grouped by growth condition, 607, 947 optimal reads (effective rate 68.69%), 639,087 reads (effective rate 71.54 %), 716,750 reads (effective rate 74.77%), and 535, 812 reads (effective rate 78.46%) were found from the open field, solar greenhouse, arched shed, and hydroponic system.

**Table 1:** Effective sequence number in different ecological compartments.

	**Phylloplane**	**Leaf endosphere**	**Stem endosphere**	**Root endosphere**	**Rhizosphere**
Total sequences	529727	652761	610660	838167	788626
Effective sequences	489118	618959	581622	475384	334513
Effective rate	92.33%	94.82%	95.24%	56.42%	42.42%

**Figure 1:**
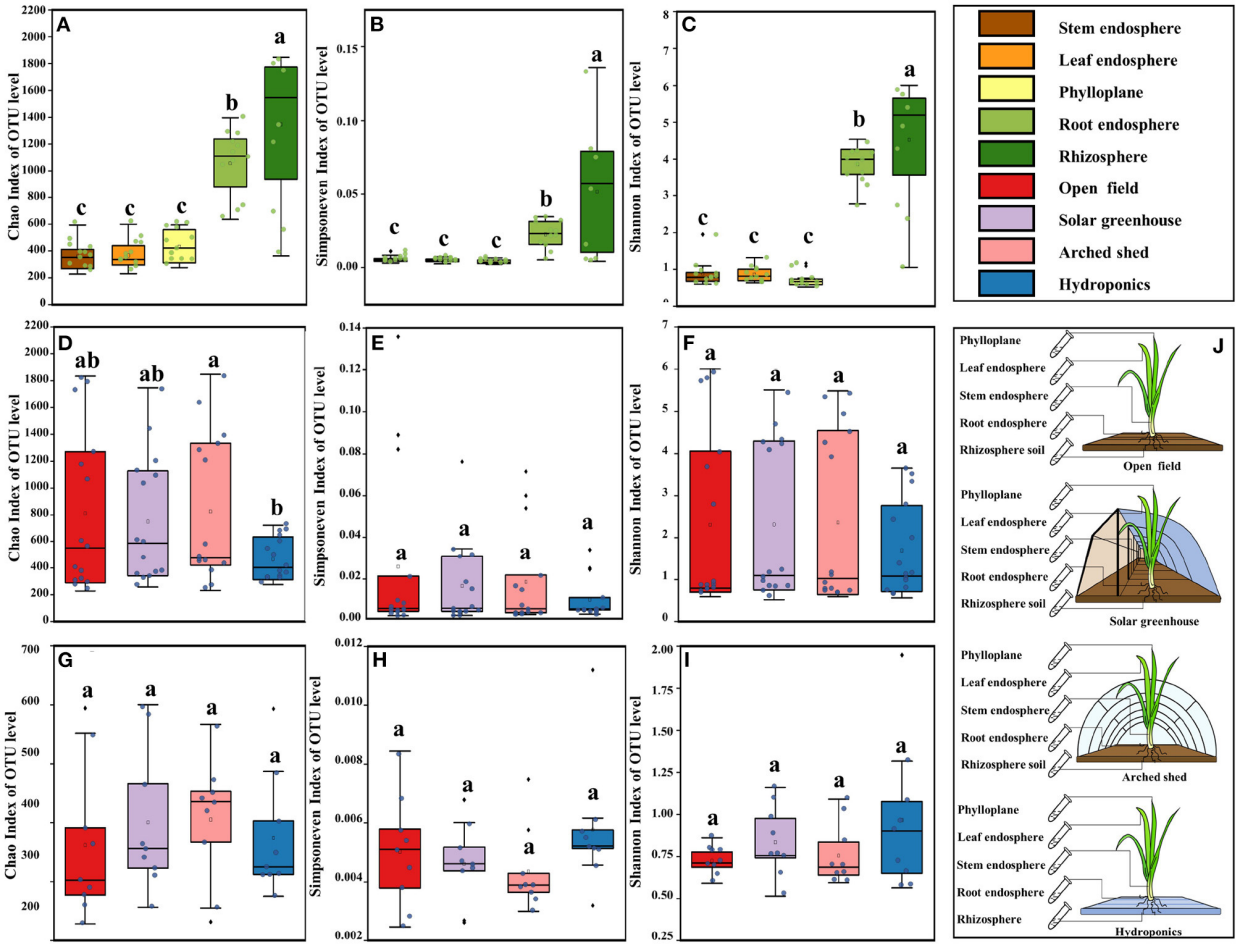
Bacterial diversity in the different ecological compartments and growth conditions. The bacterial alpha diversity in the different ecological compartments: **(A)** Chao index, **(B)** Simpson evenness index, **(C)** Shannon index. Bacterial alpha diversity in the different growth conditions: **(D)** Chao index, **(E)** Simpson evenness index, **(F)** Shannon index. Bacterial alpha diversity in aboveground ecological compartment in different growth conditions: **(G)** Chao index, **(H)** Simpson evenness index, **(I)** Shannon index, **(J)**. Diagram of sampling in the different ecological compartments and growth conditions. The horizontal bars within boxes represent medians. The tops and bottoms of boxes represent the 75th and 25th percentiles, respectively. The upper and lower whiskers extend to 1.5 × the interquartile range from the upper edge and lower edge of the box, respectively. Different letters indicate differences between groups (*P* < 0.05, ANOVA, Tukey-HSD test). The numbers of replicated samples in this figure are as follows: in different ecological compartments, *n* = 12, in different growth conditions, *n* = 15, root and rhizosphere in different growth conditions, *n* = 6.

The number of sequences was then standardized to 20,112 (minimum number of sequences among samples) for each sample, and 3,835 OTUs were clustered with 97% similarity, with a coverage index above 98.8%. Rarefaction curves (Figure 1) showed that the number of species detected from 10,000 sequences tended to be saturated, indicating that most microbial populations were covered in the sequencing results, which could thus accurately reflect the community structure of endophytic and rhizospheric microorganisms. The data described above showed that this method was suitable for the prediction and analysis of the composition and functions of bacterial populations associated with Chinese chives in different ecological compartments and growth conditions.

### 3.2. Differences in the Diversity of Bacterial Communities Associated With Chinese Chives Among Ecological Compartments and Growth Conditions

#### 3.2.1. Differences in Alpha Diversity of Bacterial Communities Associated With Chinese Chives in Different Ecological Compartments and Growth Conditions

The alpha diversity of bacteria in the phylloplane, leaf endosphere, stem endosphere, root endosphere, and rhizosphere was analyzed with the Chao index, Shannon index, and Simpson evenness index (Figures 1A–C). Compared with the belowground compartments (root endosphere and rhizosphere), diversity was low in the aboveground compartments (phylloplane, leaf endosphere, and stem endosphere), the richness and evenness were poor, and there was no significant variation among the three compartments, indicating that the diversity of bacterial communities in the aboveground compartments was uniform. Bacterial diversity was significantly higher in root endosphere and rhizosphere than in the aboveground compartments, and the richness and evenness were also significantly different (*P* < 0.001).

In the analysis of alpha diversity of bacteria in the open field, solar greenhouse, arched shed and hydroponic system, Chao, Shannon and Simpson evenness indexes were also included (Figures 1D–F). The bacterial diversity index of each growth condition was high, and the evenness was low, but it was not significantly different. The richness index of bacteria in hydroponic environment was significantly lower than that in arched shed (*P* < 0.05). These results showed that ecological compartment and growth condition had significant indigenous effects on the alpha diversity index of microbial groups, and confirmed that the effect of ecological compartment on bacterial community was higher than that of growth condition on bacterial community. Through the analysis of bacterial diversity in different ecological compartments and growth conditions, there are significant differences between underground ecological compartments in different growth conditions due to different cultivation substrates. We are more curious about whether the growth condition has an impact on the aboveground ecological compartment. Therefore, we analyzed the differences in alpha diversity of microbial bacterial communities in the aboveground ecological compartments of Chinese chives among growth conditions (Figures 1G–I). Differences in bacterial alpha diversity between aboveground ecological compartments but not significant in different growth conditions (*P* > 0.05). In the hydroponic environment, the bacterial richness and diversity in the aboveground ecological compartments were high but the evenness was low. The bacterial diversity and richness in the aboveground ecological compartments in the solar greenhouse environment were higher than those in the arched shed environment, but the evenness was lower than that in the arched shed environment. Therefore, although the effect of growth condition on the bacterial diversity of aboveground ecological compartments was not significant, there were some effects.

#### 3.2.2. Differences in Beta Diversity of Bacterial Communities in Different Niches and Growth Conditions

**Figure 2:**
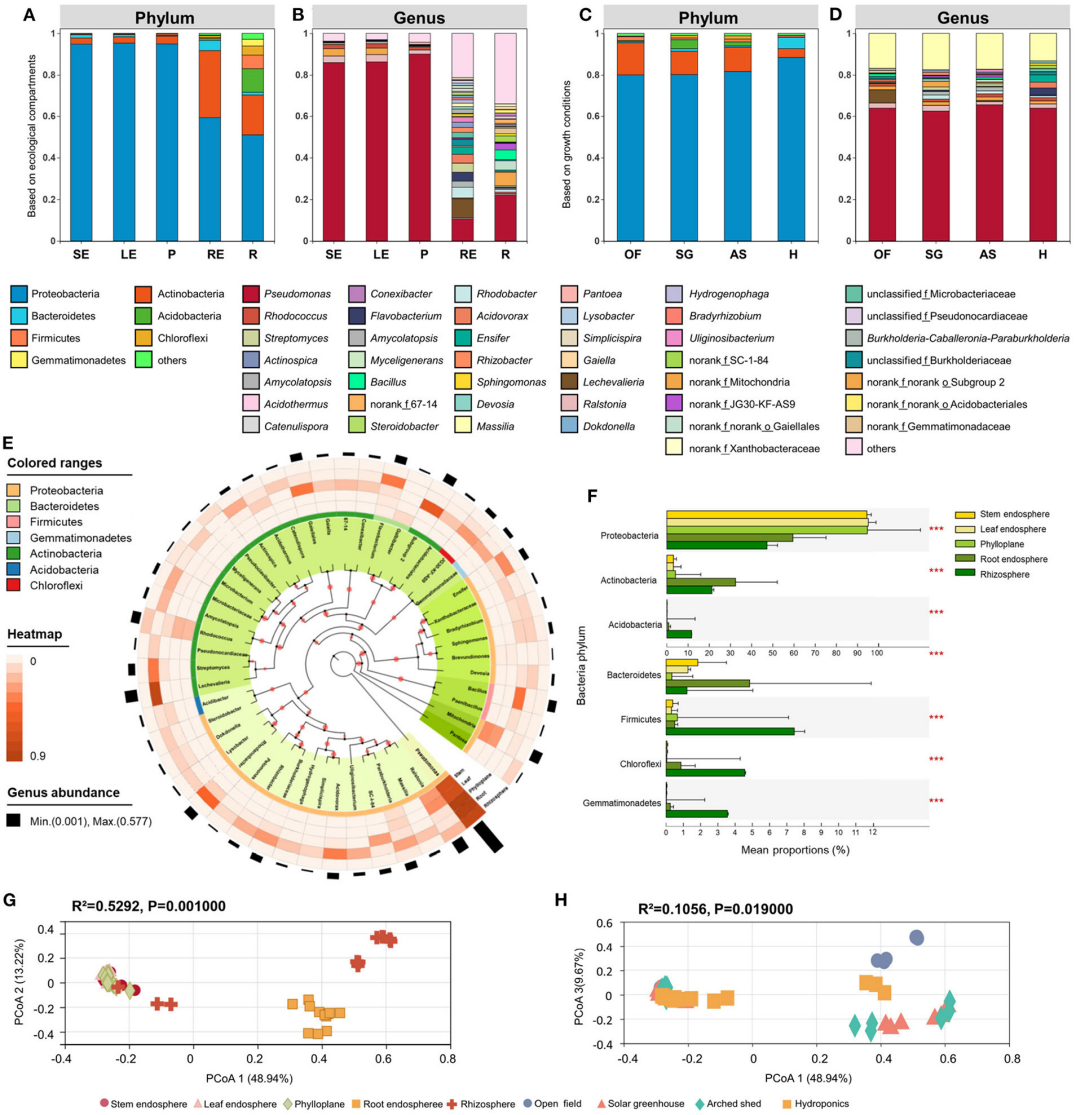
Bacterial community composition in different ecological compartments and growth conditions. The bacterial composition at the phylum **(A)** and genus **(B)** levels in different ecological compartments. SE, stem endosphere; LE, leaf endosphere; P, phylloplane; RE, root endosphere; R, rhizosphere. The bacterial composition at phylum **(C)** and genus **(D)** levels in different growth conditions. OF, open field; SG, solar greenhouse; AS, arched shed; H, hydroponics. **(E)** Bacterial phylogenetic tree. We selected the top 50 bacteria with relative abundance to construct the system tree. Visualization was performed on the iTOL website (https://itol.embl.de/). The colors in the inner circle represents the specific bacterial phyla. Four outer circles represent four different groups, and the height shows the abundance of genera. **(F)** Significant differences in bacterial phyla among different growth conditions. Bacterial beta diversity PCoA based on Bray-Curtis distance in ecological compartments, **(G)** and different growth conditions, **(H)**. ^*^P ≤ 0.05, ^**^P ≤ 0.01, ^***^P ≤ 0.001.

Analysis of beta diversity with PCoA showed separate clustering of the root endosphere and rhizosphere groups (Adonis test. *P* = 0.001) (Figure 2G). We further analyzed the bacterial content in the root endosphere and rhizosphere. Diversity, richness and evenness of rhizospheric bacteria were significantly higher than for those in roots (*P* < 0.05). Additionally, bacterial beta diversity showed a specific clustering among root endosphere, rhizosphere, and aboveground compartment (stem endosphere, leaf endosphere, phylloplane) samples (Adonis test, *P* = 0.001). This indicates significant differences in bacterial composition among the root endosphere, rhizosphere, and other ecological compartments.

### 3.3. Composition of Bacterial Communities Associated With Chinese Chives in Different Ecological Compartments and Growth Conditions

#### 3.3.1. Differences in Endophytic Bacterial Components in Different Ecological Compartments of Chinese Chives

To study the effect of ecological compartment on bacterial community structure, we analyzed the group differences among different ecological compartments and compared the bacterial communities of the phylloplane, leaf endosphere, stem endosphere, root endosphere, and rhizosphere, including comparisons at the phylum and genus level. The differences in abundance among Bacteroidetes, Firmicutes, Chloroflexi, Gemmatimonadetes, Proteobacteria, Actinobacteria, and Acidobacteria were extremely significant (*P* < 0.001) at the phylum level (Figure 2F), while at the genus level, the abundance differences among *Pseudomonas*, *Ralstonia*, *Lechevalieria*, *Rhodanobacter*, *Rhodococcus*, *Bacillus*, and *Flavobacterium* were extremely significant (*P* < 0.001).

Consistent with findings of previous studies, Proteobacteria was the dominant phylum among the identified OTUs, and among the five ecological compartments, Proteobacteria was most dominant in the leaf endosphere (95.35% relative abundance) and least abundant in the rhizosphere (51.00%) but was still the most dominant phylum in the rhizosphere. Actinobacteria ranked second in relative abundance, with the highest distribution in root endosphere (32.46%) and the lowest distribution in stem endosphere (29.40%). *Pseudomonas* was the most important genus of bacteria among the identified OTUs (Figures 2A,E). *Pseudomonas* was most dominant in the phylloplane, accounting for 90.04% of bacterial genera, accounted for over 85% in the leaf endosphere and stem endosphere, and was least abundant in the root endosphere, accounting for 10.47%. Richness values of aboveground ecological compartments were lower than those of belowground ecological compartments: a total of 716 bacterial genera were detected in the rhizosphere, which exceeded by 100 those in the aboveground compartments. At the genus level, the community composition in aboveground compartments (phylloplane, leaf endosphere, and stem endosphere) was significantly different from that of belowground compartments, and the abundances of *Lechevalieria*, *Rhodanobacter*, *Flavobacterium*, and *Burkholderia* − *Caballeronia* − *Paraburkholderia* in belowground compartments were higher than values in aboveground compartments (Figure 2B). At the phylum level, the abundances of Proteobacteria (59.26%), Actinobacteria (32.46%), and Bacteroidetes (4.93%) in the root endosphere were higher than those in the rhizosphere group. At the genus level, differences between the rhizosphere and root endosphere were small, but *Lechevalieria* (9.10%), *Rhodanobacter* (5.14 %), and *Flavobacterium* (4.32%) were more abundant in the root endosphere than in the rhizosphere.

#### 3.3.2. Differences in Phylloplane, Rhizosphere and Endophytic Bacterial Components in Chinese Chives Under Different Growth Conditions

Proteobacteria was the main phylum under all growth conditions. Relative abundance of Proteobacteria varied little among growth conditions; the highest percentage was 88.42% in the hydroponic environment, and the lowest was 80.2% in the field. Actinobacteria was the second most abundant bacterial phylum, but its distribution pattern was opposite to that of Proteobacteria. Actinobacteria was least abundant in hydroponics (4.26%) and most abundant in the field (15.48%). At the phylum level, Acidobacteria and Bacteroidetes differed significantly in relative abundance among groups (*P* < 0.001). Compared with the three groups with soil as the cultivation substrate, the relative abundances of Proteobacteria (88.42%) and Bacteroidetes (5.40%) in the hydroponic group were significantly elevated, but the relative abundance of Actinobacteria (4.26%) was significantly lower (Figure 2C).

At the genus level, the differences in abundance of *Lechevalieria*, *Burkholderia* − *Caballeronia* − *Paraburkholderia*, *Streptomyces*, and *Ensifer* among groups were extremely significant (*P* < 0.001). *Pseudomonas* was the dominant genus among these, with the greatest abundance in the arched shed (65.45%) and the lowest abundance in the solar greenhouse (62.41%). The relative abundance of *Burkholderia* − *Caballeronia* − *Paraburkholderia* in solar greenhouse and arched shed was significantly higher than that in open field and hydroponic environments. The abundance of *Lechevalieria* (6.37%) in the open field was higher than numbers under the other three conditions, but the abundance of *Rhodanobacter* (0.05%) was significantly reduced. The abundances of *Flavobacterium* (3.61%), *Acidovorax* (2.83%), *Ensifer* (3.48%), and *Rhizobacter* (1.44%) were significantly higher in the hydroponics (Figure 2D).

### 3.4. Specific Bacterial Communities and Differences in Bacterial Communities in Different Ecological Compartments and Growth Conditions

**Figure 3:**
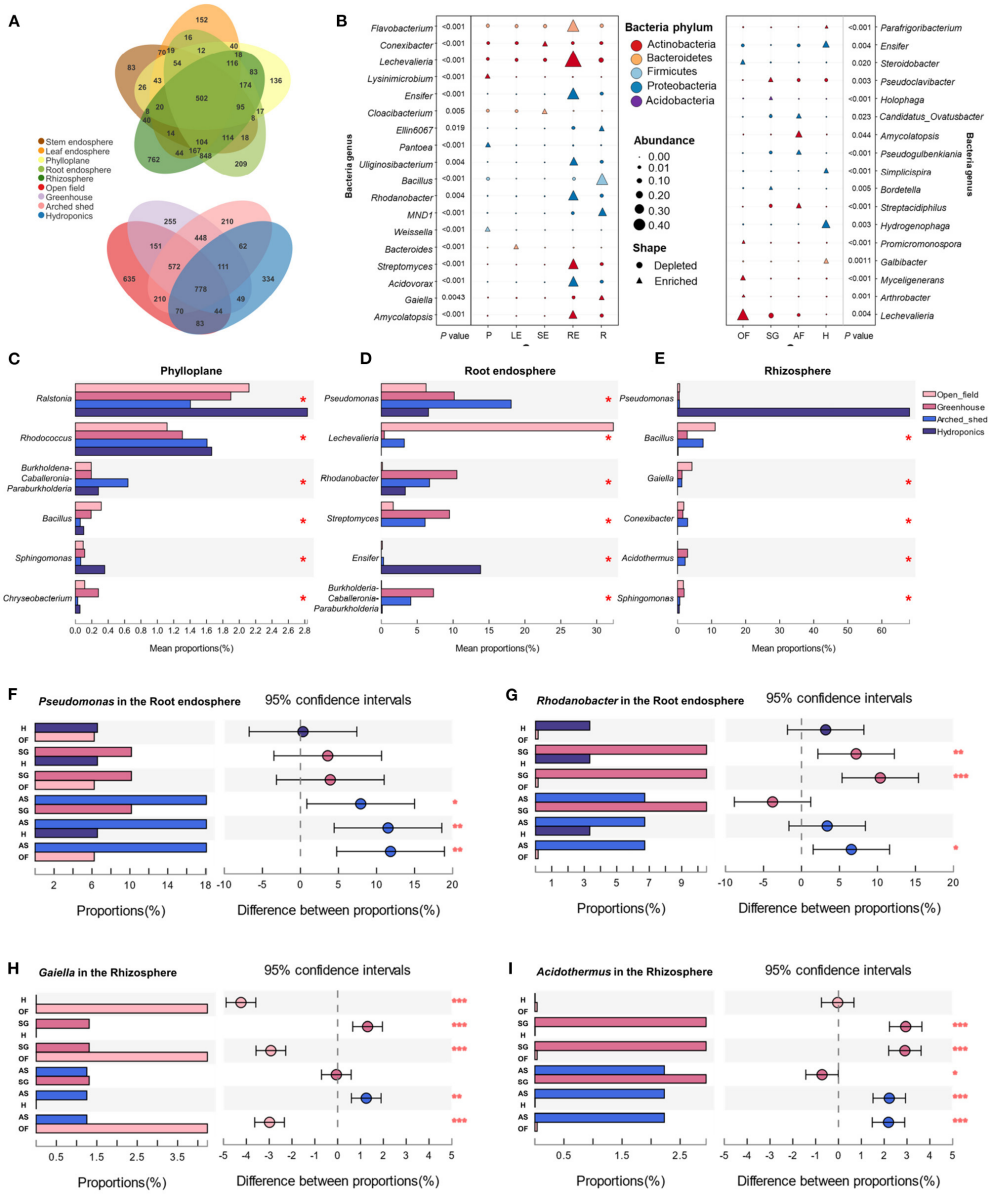
Specific bacterial communities in different ecological compartments. **(A)** UpSet plot shows the OTU count of each group. **(B)** Indicator species in ecological compartments. The bacterial abundance at the genus level was analyzed. The shape of the point represents the enrichment or deletion of OTUs in the population, and the size of the point represents the abundance of OTUs. SE, stem endosphere; LE, leaf endosphere; P, phylloplane; RE, root endosphere; R, rhizosphere. Differences in bacterial genera in the phylloplane, **(C)** root endosphere, **(D)**; and rhizosphere, **(E)** under growth conditions. Comparison and analysis of OTU abundances of *Pseudomonas* **(F)** and *Rhodanobacter* **(G)** in the root endosphere and *Gaiella* **(H)** and *Acidothermus* **(I)** in the rhizosphere among different growth conditions. OF, open field; SG, solar greenhouse; AS, arched shed; H, hydroponics. ^*^P ≤ 0.05. ^**^P ≤ 0.01. ^***^P ≤ 0.001.

First, specific bacterial communities were analyzed in different ecological compartments. There were 4,012 OTUs in different compartments and 502 OTUs shared by all groups, but different ecological compartments also featured unique bacterial communities. A total of 762 OTUs existed only in the rhizosphere, 209 OTUs existed only in roots, and the number of unique OTUs in the stem was the lowest at 83. There were 848 OTUs in the root endosphere and rhizosphere. In addition, although the numbers of OTUs in the phylloplane, leaf endosphere, and stem endosphere were similar, there were only 43 shared OTUs, so bacterial colonization was not uniform among aboveground ecological compartments. These data indicate that different ecological compartments have a greater impact on OTUs (Figure 3A).

We further analyzed the group-specific bacterial genera in each ecological compartment and growth condition and determined the indicator species in each ecological compartment, namely, the bacterial genera that were significantly enriched in one ecological compartment or growth condition and not present in other groups (Figure 3B). We found that *Flavobacterium*, *Rhodanobacter*, *Streptomyces*, *Acidovorax*, *Amycolatopsis*, *Lechevalieria*, *Uliginosibacterium*, and *Ensifer* were enriched in the root endosphere, and *Lechevalieria* could be used as a better indicator of root endosphere ecological communities. *Bacillus*, *Gaiella*, *Conexibacter*, *Ellin*6067, and *MND*1 were enriched in the rhizosphere, and *Bacillus* can be used as a better indicator for the identification of rhizospheric bacterial communities. *Conexibacter* and *Cloacibacterium* were abundant in the stem endosphere and could be used as indicator bacteria for stem endosphere ecological communities. *Lysinimicrobium*, *Pantoea*, and *Weissella* were enriched in phylloplane and can be used as indicators to identify bacterial genera in phylloplane ecological communities. *Bacteroides* only enriched in the leaves can be used as indicator bacteria to identify the ecological compartments on the leaf endosphere. Compared with the above ecological compartments, there were fewer bacteria enriched in the leaf endosphere because most of the bacteria enriched in the leaf endosphere were also enriched in the stem endosphere.

In addition, to characterize bacteria in ecological compartments where external bacterial exchange is more frequent, we compared the bacterial genera in the phylloplane, root endosphere, and rhizosphere under different growth conditions. We found that *Ralstonia* and *Rhodococcus* were significantly more numerous in the hydroponic environment (Figure 3C). *Pseudomonas* was more likely to colonize the root endosphere in the arched shed environment, while *Lechevalieria* in the root endosphere was significantly more abundant in the open field environment (Figure 3D). Because the hydroponic environment is different from the cultivation substrates in the other three environments, the bacterial differences in the rhizosphere are mainly reflected in the hydroponic environment. Pseudomonas in the rhizosphere of hydroponic culture is significantly higher than that in other environments (Figure 3E).

Many bacteria were enriched in the belowground compartments. We wanted to further understand which bacterial genera were highly abundant in the root endosphere and rhizosphere under different growth conditions. Therefore, we compared the differences in bacterial genera in the root endosphere and rhizosphere under different growth conditions. We found that *Pseudomonas* was more enriched in the hydroponic root endosphere (Figure 3F), while *Rhodanobacter* was more abundant in the solar greenhouse and arched shed but was absent in the open field (Figure 3G). In contrast, *Gaiella* was highly enriched in the rhizosphere (Figure 3H), and *Acidothermus* was significantly more numerous in the solar greenhouse and arched shed (Figure 3I). This indicates that bacteria will exhibit specific enrichment patterns in different ecological compartments and growth conditions. The same ecological compartment in different growth conditions has a great influence on bacterial colonization, which is more obvious in underground compartments.

### 3.5. Interaction Patterns and Prediction of Metabolic Pathways of Bacterial Communities Associated With Chinese Chives Under Different Ecological Compartments and Growth Conditions

#### 3.5.1. Differences in Interaction Patterns of Bacteria Associated With Chinese Chives Under Different Growth Conditions

**Figure 4:**
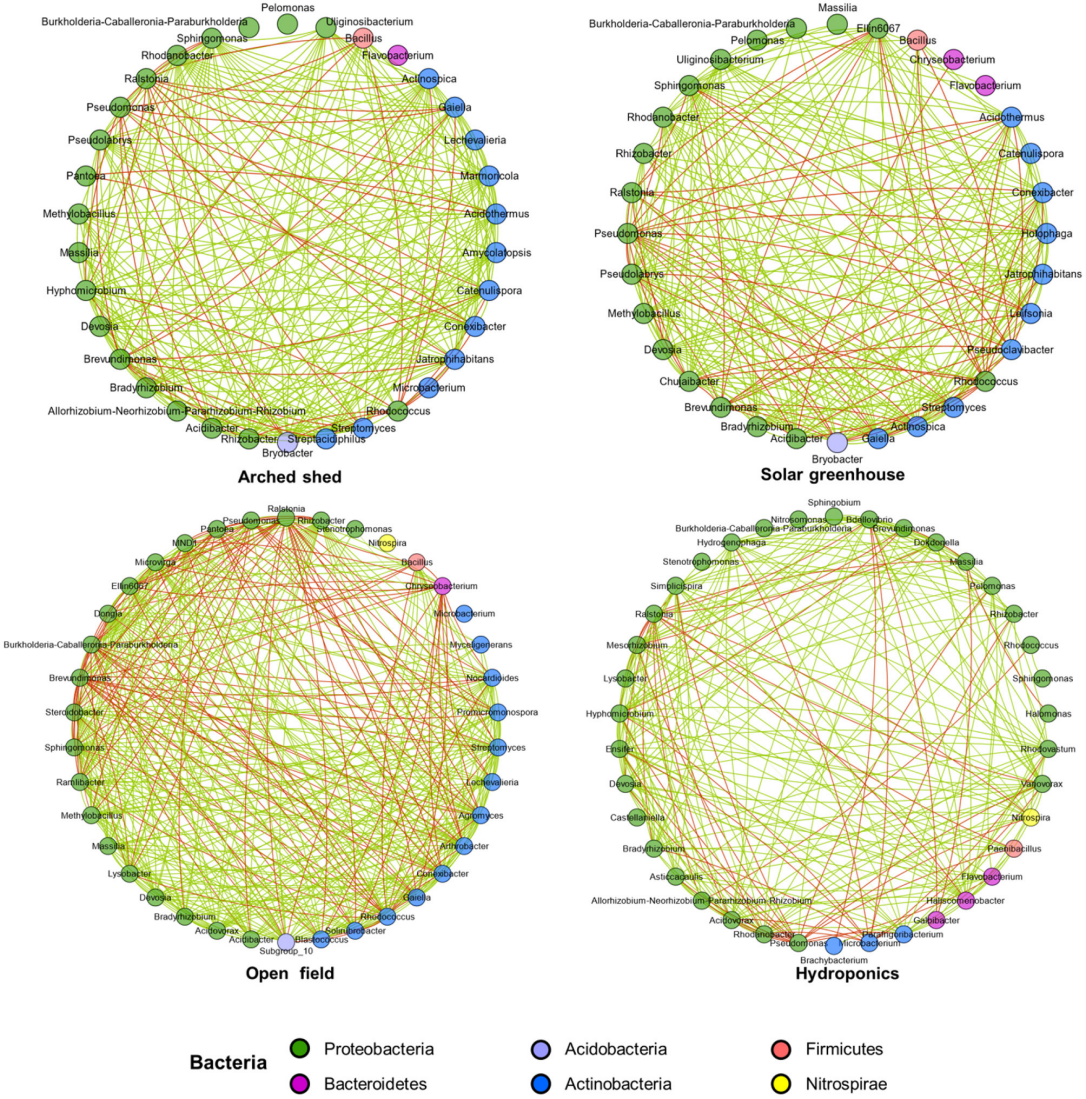
Patterns of interaction of bacteria in different growth conditions. Spearman's test was used to analyze the interactions among the top 50 bacterial OTUs by abundance. Only a significant correlation (*P* < 0.05) is shown as a line. Line color indicates positive (green) or negative (red) correlation depending on the Spearman correlation coefficient. These nodes represent microbial genera, and their colors represent microbial phyla.

Interactions between bacteria are very important for maintaining microbial homeostasis. To explore the interaction networks among bacterial phyla and bacterial genera under different growth conditions, we selected the top 50 bacterial genera for correlation analysis. In the hydroponic environment, although interactions between bacteria were weaker than under the other three growth conditions, there were still a greater number of bacterial interactions, and mutual promotion outweighed inhibition, which was also shown in other growth conditions. In the hydroponic environment, a few Actinobacteria (including *Microbacterium* and *Brachybacterium*) had negative interactions with Proteobacteria (Figure 4, Supplementary Figure S2). The interactions of *Pseudomonas* and *Ralstonia* in Proteobacteria with other bacteria were mostly negative, while the interactions of *Flavobacterium*, *Galbibacter* and *Haliscomenobacter* in Bacteroidetes with other bacteria were mostly positive and frequent. We observed that there were more genera with negative interactions with *Ralatonia* in different growth conditions, such as *Lysobacter*, *Variovorax*, *Hyphomicrobium*, and *Mesorhizobium*.

It is worth noting that in the open field environment, negative interactions between bacteria are more frequent, and there are more competitive relationships. Negative correlations of *Chryseobacterium* with Proteobacteria, Actinobacteria, and Acidobacteria were more frequent in the open field. For close interactions in the open field, *Brevundimonas* showed a strong negative interaction with *Bacillus* and *Conexibacter*, and there was also a negative correlation between *Rhodococcus* and *Agromyces* in Actinobacteria. The interactions of *Rhizobacter* and *Stenotrophomonas* in Proteobacteria with other bacteria were mostly positive.

Compared with the open field, the positive and negative interactions between bacteria in the artificial environment were reduced. Therefore, the original relevant models established by some bacteria were interrupted, and new interactions were reconstructed in the artificial environment. Although the arched shed and solar greenhouse were similar sheltered structures, according to the grid of interactions between bacteria, the arched shed seemed to be more conducive to positive interactions between bacteria.

#### 3.5.2. Analysis of the Bacterial Interaction Network in Chinese Chives Between Different Ecological Compartments

**Figure 5:**
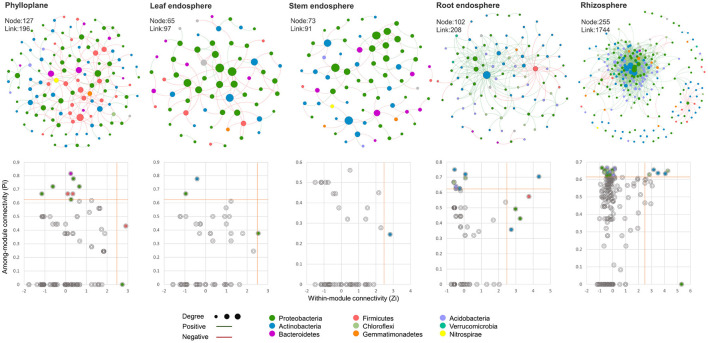
The correlation networks of bacteria in different ecological compartments. One node represents an OTU, and the size of the node is determined by the degree. The larger the degree is, the larger the corresponding node size is, and the color of the node represents the microbial phylum which the OTU belongs. Line color indicates positive (green) or negative (red) correlation.

To further analyze the effect of ecological compartments on the interaction between bacteria in Chinese chive, we selected OTUs with more than 6 OTUs in each sample to carry out the analysis of the bacterial community cooccurrence network and the calculation of the topological characteristic index in MENA. In this study, the modular index of aboveground compartments was higher than 0.60 (Table 2) and much higher than that of underground compartments, indicating that microorganisms in aboveground compartments had modular structure, and ecological compartments had a significant indigenous effect on the modular of the microbial interaction network. The average clustering coefficient and network density reflect the degree of aggregation of nodes and the complexity of the network structure in the co-occurrence network. In this analysis, there were great differences between rhizosphere ecological compartments and other compartments. The average clustering coefficient and average degree of the rhizosphere were high, and the positive interaction relationship was strong, but the network density was low. This indicates that the aggregation of bacterial communities between underground compartments is poor, the positive correlation between microorganisms is strong, and other functions are more complex. This is consistent with the conclusion that underground compartments have higher species richness and community diversity than aboveground compartments. To identify the microbial groups that play key nodes (OTUs) in the interaction network, we calculated the within-module connectivity (Zi) and among-module connectivity (Pi) of each network node (OTUs). All networks have higher modularity characteristics than their random networks (Table 2), indicating that it is reasonable to divide these networks into modules. According to the topological characteristics of nodes, node attributes can be divided into the following four types: nodes with high connectivity within the module, Modulehubs (Zi>2.5); nodes with high connectivity between the two modules, Connectors (Pi>0.62); nodes with high connectivity throughout the network, Networkhubs (Zi <2.5 and Pi>0.62) and nodes without high connectivity within and between modules, Peripherals (Zi>2.5 and Pi <0.62). Except for peripherals, all nodes of three types belong to the key nodes (OTUs). It can be found in the network we construct (Figure 5) that the vast majority of nodes in each network belong to peripherals, and only a few nodes have a high degree of connection. Compared with the aboveground compartment microbial networks, the rhizosphere and root endosphere microbial networks of Chinese chives have more Modulehubs and Connectors, and Networkhubs were also detected in the rhizosphere and root endosphere microbial networks. These phenomena consistently indicate that there are multiple key microbial groups in the microbial community of Chinese chive underground compartments that dominate the occurrence of more complex interspecific interactions. We show the gate phylum level (Figure 5) and its genus classification level (Table 2) of all network nodes to determine the inherent attributes of key network nodes. The results showed that more than half of Modulehubs occupied the dominant relative abundance group, while most connectors mainly existed in lower relative abundance, which was more obvious in the underground ecological compartment. Proteobacteria are the most prominent key groups except for the microbial network of the stem endosphere, and the nodes that play a key role in the microbial network of the stem endosphere are not the highest relative abundance of Proteobacteria in the stem endosphere but the highest relative abundance of Actinobacteria in underground ecological compartments. In addition to Proteobacteria, Acidobacteria, and Actinobacteria are the main key nodes in rhizosphere and root endosphere microbial networks, respectively. Chloroflexi, Firmicutes, Gemmatimonadetes, Verrucomicrobia, and Armatimonadetes also play a key role in underground ecological compartments.

**Table 2:** Topological properties of the empirical ecological networks at different ecological compartments in comparison to the random networks.

**Groups**	**Empirical network indexes**					**Random networks indexes**		
	**Average degree (avgK)**	**Density (D)**	**Average clustering coefficient (avgCC)**	**Average path distance (GD)**	**Modularity**	**Average clustering coefficient (avgCC)**	**Average path distance (GD)**	**Modularity**
P	3.087	0.024	0.122	4.58	0.638	0.023 ± 0.011	4.135 ± 0.105	0.563 ± 0.012
LE	2.985	0.047	0.087	4.223	0.626	0.040 ± 0.019	3.697 ± 0.140	0.526 ± 0.017
SE	2.493	0.035	0.131	5.56	0.725	0.027 ± 0.018	4.406 ± 0.219	0.617 ± 0.017
RE	4.078	0.04	0.016	3.137	0.402	0.211 ± 0.029	2.806 ± 0.058	0.395 ± 0.009
R	13.678	0.073	0.155	3.157	0.132	0.335 ± 0.010	2.464 ± 0.017	0.094 ± 0.002

*P, phylloplane; LE, leaf endosphere; SE, stem endosphere; RE, root endosphere; R, rhizosphere*.

#### 3.5.3. Prediction of the Metabolic Pathways of Endophytic Bacteria Associated With Chinese Chives in Different Ecological Compartments and Growth Conditions

To further predict and analyze the functions of endophytic bacteria in different growth conditions, we used the sequencing results and the SILVA database to annotate the functions of microbial communities in different ecological compartments and growth conditions through the Tax4fun package of R. The metabolic functions of all microorganisms were mainly focused on carbohydrate metabolism, amino acid metabolism, signal transduction, and membrane transport within metabolism, as well as environmental information processing. There were significant group differences among ecological compartments, which were mainly differences between the aboveground and belowground compartments. In most metabolic functions, the differences between the stem endosphere, leaf endosphere, and phylloplane of the aboveground compartment were small, and the differences between the root endosphere and rhizosphere were only significant for carbohydrate metabolism and energy metabolism. Most of the secondary pathways of genetic information processing and metabolism, such as amino acid metabolism, metabolism of cofactors and vitamins, energy metabolism, xenobiotic degradation and metabolism, lipid metabolism, and metabolism of terpenoids and polyketides, were more prevalent in the belowground compartments than in the aboveground compartments, but the secondary pathways of environmental information processing and cellular processes were less prevalent belowground. It is worth noting that cell motility within cellular processes was significantly lower in the root endosphere than in other compartments.

In contrast to the significant differences in the prevalence of group pathways among ecological compartments, differences in growth conditions had little effect on microbial metabolic pathways. Differences observed were mainly present between hydroponic and other growth conditions. Infectious microbial pathogens were more abundant in the hydroponic system than under other growth conditions, while amino acid metabolism was significantly lower in the hydroponic environment than in other growth conditions. The differences between hydroponic and other growth conditions were mainly reflected in organismal systems. The environmental adaptation pathway was significantly more prevalent in the hydroponic system than in the other groups, but endocrine, digestive, and nervous functions were significantly depressed in the hydroponic system (*P* < 0.01).

Among all microbial metabolic pathways, a total of 277 tertiary metabolic pathways were detected, and group difference tests were performed on 15 tertiary metabolic pathways under carbohydrate (Supplementary Figure S3) metabolism, the secondary metabolic pathway with the greatest prevalence. We found that the variation was mainly manifested in differences between the aboveground and belowground compartments, and differences among the aboveground compartments were small. The levels of citrate cycle (TCA cycle) metabolism, butanoate metabolism, inositol phosphate metabolism, propanoate metabolism, and glyoxylate and dicarboxylate metabolism were significantly lower than those in the belowground compartments (*P* < 0.05).

## 4. Discussion

In this study, the specific differences in bacteria in different ecological compartments and growth conditions were described, and their metabolic pathways were predicted. The results showed that bacterial communities differed significantly among plant ecological compartments, and modes of interaction and function were also significantly different among groups.

### 4.1. Diversity of Chinese Chive Bacteria Was Affected by Ecological Compartment and Growth Condition

The Chinese chive microbial community consists of multiple ecological compartments (phylloplane, leaf endosphere, stem endosphere, root endosphere, and rhizosphere), each with different microbial compositions. Within the study, we found that the diversity and richness of bacteria in the stem endosphere, leaf endosphere and phylloplane were significantly lower than those in the root endosphere and rhizosphere (Figure 1). Plants often distribute many nutrients to roots (Bulgarelli et al., 2012) during their growth and release large amounts of nutrients and energetic compounds, such as monosaccharides, polysaccharides, organic acids, phenolic compounds, amino acids, and proteins (Bais et al., 2006), to the surrounding environment through their roots, and these resources attract large aggregations of microbes. In rhizosphere secretions, compounds belonging to the chemical class of phenolics and terpenoids have strong external antibacterial and antifungal properties. Among them, phenolic metabolites can also effectively attract some soil-borne microorganisms and have beneficial effects on local soil microbial communities. Phenolic metabolites alter internal and external plant environments and various ecological compartments and promote the colonization and growth of specific bacteria. However, this phenomenon was not obvious in the hydroponic environment. The overall bacterial diversity and richness in the hydroponic environment were low, and differences in alpha diversity among the plant ecological compartments were not significant. Diversity was lower in the rhizospheric water, and diversity and evenness in the stem endosphere, leaf endosphere, and phylloplane in the hydroponic system tended to be more consistent. These results are consistent with the research findings of Stouvenakers (Stouvenakers et al., 2020). Microbial diversity and interaction among ecological compartments of plants are related to their ability to resist external disturbance (Kusstatscher et al., 2019; Xie et al., 2021). In this study, the alpha diversity in the Chinese chive rhizosphere was significantly higher than that in other ecological compartments, the rhizosphere microbial network average degree and average clustering coefficient were higher than those in other ecological compartments, and the interaction of the microbial network was more complex. This may be related to the direct contact between the rhizosphere and soil (nutrient solution), the generation of a bacterial screening mechanism and the inhibitory effect of the rhizosphere (Reinhold Hurek et al., 2015; Itumeleng et al., 2020). Inhibition may result from (trace) nutrient competition, antibiotic compounds, production of decomposing enzymes, consumption (Lugtenberg and Kamilova, 2009; Doornbos et al., 2012) of pathogen-stimulating compounds or other pathways. The different growth conditions we sampled had little effect on the diversity of microorganisms, which might be because although the air temperature and humidity were significantly different under different growth conditions, fertilization and management methods were similar. Therefore, the differences in soil physical and chemical properties among open fields, solar greenhouses and arched sheds were small, and the impact on microorganisms was small.

### 4.2. Ecological Compartments and Growth Conditions Create Different Microbial Community Structures and Succession Patterns

**Figure 6:**
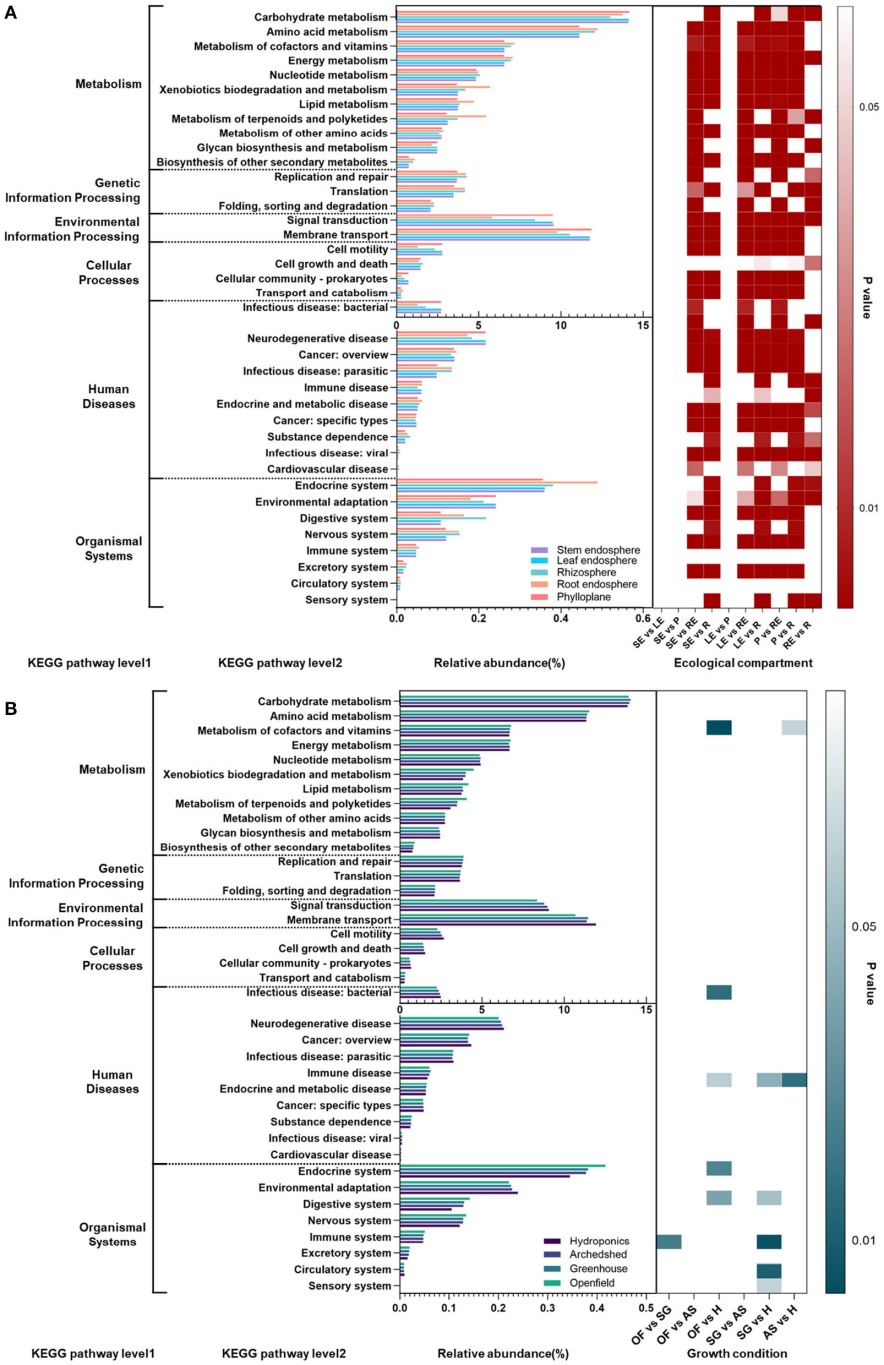
**(A)** Prediction and analysis of bacterial metabolic pathways in different ecological compartments and different growth conditions. SE, stem endosphere; LE, leaf endosphere; P, phylloplane; RE, root endosphere; R, rhizosphere. **(B)** The bar chart shows the abundance of each group, and the different levels of metabolism are separated by dark gray lines. The heatmaps on the right side indicate significant pairwise differences in abundance and are plotted based on the P values. OF, open field; SG, solar greenhouse; AS, arched shed; H, hydroponics.

Proteobacteria were dominant in the stem endosphere, leaf endosphere and phylloplane (Figure 2A), which was consistent with the findings of previous studies (Wang et al., 2020c). Proteobacteria were significantly negatively correlated with the abundance of Actinobacteria, the second most abundant phylum (Figures 2A,C, Supplementary Figure S2), which was one of the most important features distinguishing aboveground and belowground ecological compartments. The genus *Pseudomonas* showing the highest abundance in Proteobacteria is consistent with the results obtained by Lugtenberg and Kamilova (2009; Zhuang et al., 2020) in the rhizosphere of *Allium* plants. *Pseudomonas* plays a more important role in the co-occurrence network of rhizospheric bacteria during the late growth stage and under favourable growth conditions for *Allium* plants, and because *Pseudomonas* is present in aboveground ecological compartments, it can be isolated and purified by culturomics for biological control of Chinese chives gray mold. Among species in this genus, *Pseudomonas* aeruginosa (Wang et al., 2020b), *Pseudomonas* chlororaphis (Cw et al., 2021), *Pseudomonas fluorescens* (Zhu et al., 2021), and *Pseudomonas* extremorientalis (Wang et al., 2019) have been successfully isolated from pear rhizosphere soil, tomato and Chinese cherry and purified and show over 85% inhibitory effects on *Botrytiscinerea*. Compared with aboveground ecological compartments, the relative abundance of *Lechevalieria* in the Actinobacteria phylum in roots and rhizosphere was high. For *Lechevalieria*, mangromicins, one of their secondary metabolites, could eliminate free radical activity (as assayed by DPPH, Takuji et al., 2014) and exhibit high inoxidizability. In addition, secondary metabolites have also been found to exert antimicrobial activity and significant inhibitory effects on *Bacillussubtilis*, *Kocuriarhizophila*, *Xanthomonascampestrispv*. *oryzae* KB88, and *Candidaalbicans* (Kimura et al., 2018). These results show that the response of microbial communities in the root endosphere to fluctuations in the growth environment was more stable than those in the rhizosphere or soil (Almario et al., 2017; Han et al., 2020; Xie et al., 2021). Bacteroidetes is an important component of the bacterial microbial community (Fernández-Gómez et al., 2013) in a normal aquatic environment (Chen et al., 2011), which is related to the requirement of a strict anaerobic environment as a growth condition for most bacterial strains in this phylum. Bacteria in Bacteroidetes can hydrolyze organic matter such as carbon polymers and proteins in aquatic environments and produce a variety of extracellular enzymes to decompose or utilize complex carbon sources (Kirchman, 2002). Therefore, Bacteroidetes may play an important role in maintaining the aquatic ecosystem balance in hydroponic Chinese chives. Moreover, the bacterial communities classified within Bacteroidetes are closely associated with the decomposition of protein organic substances (Ding et al., 2019), and the conversion of these organic substances is an important aspect of carbon cycling inside plants and in the rhizosphere. Therefore, the metabolic functions of microorganisms, such as signal transduction, membrane transport and metabolism of other amino acids, are more highly expressed in hydroponics (Figure 6) than under other growth conditions.

### 4.3. Different Colonization Patterns of Bacteria in Ecological Compartments

There are vertical changes in the establishment of plant endophytic microorganisms and phyllosphere and rhizosphere microorganisms, and the structure of microbial community changes with plant ecological compartments. In this study, the dynamic patterns of phylloplane, rhizosphere and endophytic microbial communities were also significantly correlated with the ecological compartment of Chinese chive (Figures 1, 2, 6). The beta diversity analysis based on OTUs revealed that the microbial community had obvious ecologica compartment division characteristics. There are both common bacterial communities and specific bacterial communities in different ecological compartments of Chinese chives. The diversity index of microbial bacteria in the rhizosphere was high, and the number of specific bacteria was large. It can be seen from the relative abundance and interaction network that most of the key nodes in the phylloplane and leaf endosphere of Chinese chives existed in the root endosphere and rhizosphere networks. As key nodes Proteobacteria and Firmicutes in the phylloplane ecological compartment, they are also key nodes in the root endosphere (Figure 5). Rhizosphere microorganisms could enter the roots of Chinese chives, and some endophytic bacteria migrated from the root endosphere of Chinese chives to the upper stems and leaves (Edwards et al., 2015; Xie et al., 2021). There were some differences in diversity, bacterial composition, interaction network, and function among the microorganisms in the phylloplane, leaf endosphere, and stem endosphere of Chinese chives (Figures 1, 2, 5, 6). This may be because the phylloplane microorganisms of Chinese chives are derived from the diffusion of endophytes in the stem endosphere and leaf endosphere, and on the other hand, the phylloplane is close to the soil (nutrient solution) and directly or indirectly contacts the microorganisms from the soil (nutrient solution) (Melotto et al., 2008), thus forming different bacterial communities from the leaf endosphere and stem endosphere. However, it can be clearly seen in the interaction network that this part of the differential bacterial communities (existing in the leaf endosphere but not in the stem endosphere) has similarities with the rhizosphere bacterial communities. For example, we observed that *Weissella* is an indicator species of Chinese chive phylloplane in our analysis of specific bacterial communities. Because *Weissella* is mostly distributed in soil or river sediments (Fusco et al., 2015), we believe that the original source of *Weissella* in leaves is soil or nutrient solution. According to our analysis of the specific bacterial communities in different ecological compartments, the number of indicator bacteria in the root endosphere was much higher than that in other ecological compartments (Figure 3B). The rhizosphere environment exists as a transition boundary between soil (nutrient solution) and plants. Similar to phyllosphere microorganisms, the microorganisms inhabiting the rhizosphere of plants are directly affected by the host plant and the soil (nutrient solution) environment. Therefore, the r root endosphere is more plant-specific (Figure 3B), which is quite different from the microorganisms in phylloplane and rhizosphere environments. In the beginning, root endosphere microorganisms are strictly regulated, and then the entry and exit of related groups are more rigorously controlled (Liu et al., 2017). The formation of rhizosphere bacterial communities mainly follows the principle of niche adaptation, while the root endosphere bacterial communities are more significantly regulated by host signals.

### 4.4. Ecological Compartment and Growth Conditions Have Significant Effects on the Complexity and Functional Expression of the Plant Microbial Network

The results showed that the cooccurrence patterns of microorganisms in each ecological compartment of Chinese chive were nonrandom (Table 2), and the nonrandom interaction pattern reflected niche sharing or synergy between microorganisms. The networks between the ecological compartments are dominated by positive correlations (Figure 5), indicating that more beneficial interactions exist or occupy similar niche spaces among different types of microorganisms. Due to the characteristics of the microbial community structure in each ecological compartment, the topological characteristics of all networks are significantly related to their corresponding bacterial species diversity. The number of nodes and connections in underground compartments is much higher than that in aboveground compartments (Figure 5), indicating that species diversity and interaction patterns in underground compartments are more visible and active. Compared with the aboveground ecological compartment microbial network of Chinese chives, there are a large number of isolated nodes that do not interact with other nodes in the underground compartment microbial network, resulting in relatively low connectivity of the rhizosphere microbial co-occurrence network (Figure 5, Table 2). This may be related to the microorganisms in the rhizosphere living in a variety of complex and separated microhabitats (Zhang et al., 2018). which usually do not have a large-scale exchange or niche sharing between different types of microorganisms. Although rhizosphere bacteria have the highest species richness, most species are not involved in the network interaction process because a large number of microbial groups in soil (nutrient solution) exist in an inactive or dormant state (Fierer and Lennon, 2011). Growth conditions have an impact on bacterial growth condition interactions and functional expression. In our study, correlations between bacterial species in different growth conditions were analyzed (Figure 4). Mutualistic and inhibitory relationships between bacteria directly affect the bacterial community structure under different growth conditions. For example, we observed genera that were negatively correlated with *Ralstonia* under different growth conditions, such as *Lysobacter*, *Variovorax*, *Hyphomicrobium*, and *Mesorhizobium*, which were effective factors for the extraction of bacteria antagonistic against bacterial wilt in Chinese chives (Wang et al., 2017b). In terms of prediction of metabolic functions, we found that the prevalence in root endosphere and rhizosphere of most metabolic pathways, such as genetic information processing and metabolism (Figure 6, Supplementary Figure S3), was higher than that in aboveground compartments, which was similar to findings of previous studies (Lian et al., 2016; Wang et al., 2020a; Shehata et al., 2021). We found that inositol phosphate metabolism was significantly more active in the root endosphere than in other ecological compartments in the intergroup difference test for the top 15 tertiary metabolic pathways, with the highest abundance in the secondary metabolic pathway carbohydrate metabolism. Inositol phosphates produced by this metabolic process are closely related to plant stress responses and induced system resistance. In an environment characterized by salt stress and drought stress, the expression of inositol phosphates in plants is upregulated (Tan et al., 2013), which is involved in the response of plants to abiotic stress. In addition, inositol phosphates can stimulate the development of immune cells, thereby stimulating plant defense systems (Sauer and Cooke, 2010).

In conclusion, we observed that the ecological compartments had significant indigenous effects on plant microorganisms, including the diversity of bacteria, the composition of bacterial communities, and the interaction network and functional expression of bacteria in different ecological compartments. Through the analysis of bacterial relative abundance composition, indicator genus, and bacterial interaction network in different ecological compartments, we found that bacteria in different ecological compartments had different assembly methods, but most of the bacteria were still from soil (nutrient solution). The growth conditions had little effect on the bacterial community. Except for the differences in bacterial community composition and functional expression between hydroponic and open fields, arched sheds and solar greenhouses had no significant effect on bacterial community structure.

## Data Availability Statement

The (raw sequence reads) data used to support the findings of this study have been deposited in the (NCBI) repository (accession: PRJNA742175).

## Author Contributions

NS data processing, picture drawing, and paper writing. YW sample collection and theoretical guidance. JC sample planting and theoretical guidance. PW sample collection. WS, PM, YD, ZJ, and YL sample planting and related experimental materials maintenance. All authors listed have made a substantial, direct, and intellectual contribution to the work and approved it for publication.

## Conflict of Interest

The authors declare that the research was conducted in the absence of any commercial or financial relationships that could be construed as a potential conflict of interest.

## Publisher's Note

All claims expressed in this article are solely those of the authors and do not necessarily represent those of their affiliated organizations, or those of the publisher, the editors and the reviewers. Any product that may be evaluated in this article, or claim that may be made by its manufacturer, is not guaranteed or endorsed by the publisher.

## References

[B1] Almario J. Jeena G. Wunder J. Langen G. Zuccaro A. Coupland G.. (2017). From the cover: pnas plus: root-associated fungal microbiota of nonmycorrhizal arabis alpina and its contribution to plant phosphorus nutrition. Proc. Natl. Acad. Sci. U.S.A. 114:E9403. 10.1073/pnas.1710455114 28973917 PMC5676915

[B2] Amato K. R. Yeoman C. J. Kent A. Righini N. Carbonero F. Estrada A.. (2013). Habitat degradation impacts black howler monkey (alouatta pigra) gastrointestinal microbiomes. Isme J. 7, 1344–1353. 10.1038/ismej.2013.16 23486247 PMC3695285

[B3] Bais H. P. Weir T. L. Perry L. G. Gilroy S. Vivanco J. M. (2006). The role of root exudates in rhizosphere interactions with plants and other organisms. Ann. Rev. Plant Biol. 57, 233–266. 10.1146/annurev.arplant.57.032905.105159 16669762

[B4] Bulgarelli D. Schlaeppi K. Spaepen S. Themaat E. V. Schulze-Lefert P. (2012). Structure and functions of the bacterial microbiota of plants. Ann. Rev. Plant Biol. 64, 807–838. 10.1146/annurev-arplant-050312-120106 23373698

[B5] Chao L. N. W. J. L. Z. H. Y. H. Yingcui G. (2019). Quality identification of shawo radish planted in greenhouse. Bull. Chin. Agron. 35, 43–46.

[B6] Chaouachi M. Marzouk T. Jallouli S. Elkahoui S. Gentzbittel L. Ben C. Djébali N. (2021). Activity assessment of tomato endophytic bacteria bioactive compounds for the postharvest biocontrol of botrytis cinerea. Postharvest Biol. Technol. 172:111389. 10.1016/j.postharvbio.2020.111389

[B7] Chen W. Zhang W. Li S. W. Wang L. Yun H. B. Wu X. K.. (2011). Features of soil cultivable microorganism quantity and diversity distribution under different alpine grassland ecosystems in qinghai-tibet plateau. J. Glaciol. Geocryol. 33, 1419–1426.

[B8] Chi J. Yang R. Wang A. (2012). Effect of wetland plant species and growth strategy on the distribution of paes and their monoester metabolites in the rhizosphere. J. Lake Sci. 24, 416–421. 10.18307/2012.0313

[B9] Courchesne F. Gobran G. R. (1997). Mineralogical variations of bulk and rhizosphere soils from a norway spruce stand. Soil Sci. Soc. America J. 61, 1245–1249. 10.2136/sssaj1997.03615995006100040034x

[B10] Cw A. Yan W. A. Lin W. B. Wf C. Xz A. Xc C.. (2021). Biocontrol potential of volatile organic compounds from pseudomonas chlororaphis zl3 against postharvest gray mold caused by botrytis cinerea on chinese cherry - sciencedirect. Biol. Control. 159, 104613. 10.1016/J.BIOCONTROL.2021.104613

[B11] Da Silveira A. P. D. Iório R. d. P. F. Marcos F. C. C. Fernandes A. O. de Souza S. A. C. D. Kuramae E. E.. (2019). Exploitation of new endophytic bacteria and their ability to promote sugarcane growth and nitrogen nutrition. Antonie van leeuwenhoek 112, 283–295. 10.1007/s10482-018-1157-y 30194506

[B12] Dai F. J. Chau C. F. (2017). Classification and regulatory perspectives of dietary fiber. J. Food Drug Anal. 25, 37–42. 10.1016/j.jfda.2016.09.006 28911542 PMC9333437

[B13] Ding S. Y. Wang L. Han-Chen X. U. Bao X. Y. Wang H. Chang Y. Q.. (2019). Bacterial community structure and function in the intestinal tracts and culture environment of sea cucumber (*apostichopus japonicus*). Chin. J. Ecol. 38, 210–220. 10.13292/j.1000-4890.201901.008

[B14] Doornbos R. F. Loon L. Bakker P. (2012). Impact of root exudates and plant defense signaling on bacterial communities in the rhizosphere. a review. Agron. Sustain. Develop. 32, 227–243. 10.1007/s13593-011-0028-y

[B15] Edwards J. Johnson C. Santos Medellín C. Lurie E. Podishetty N. K. Bhatnagar S.. (2015). Structure, variation, and assembly of the root-associated microbiomes of rice. Proc. Natl. Acad. Sci. U.S.A. 112, E911–E920. 10.1073/pnas.1414592112 25605935 PMC4345613

[B16] Emami S. Alikhani H. A. Pourbabaee A. A. Etesami H. Motasharezadeh B. Sarmadian F. (2020). Consortium of endophyte and rhizosphere phosphate solubilizing bacteria improves phosphorous use efficiency in wheat cultivars in phosphorus deficient soils. Rhizosphere 14:100196. 10.1016/j.rhisph.2020.100196

[B17] Fernández-Gómez B. Richter M. Schüler M. Pinhassi J. Acinas S. G. González J.. (2013). Ecology of marine bacteroidetes: a comparative genomics approach. Isme J. 7, 1026–1037. 10.1038/ismej.2012.169 23303374 PMC3635232

[B18] Fierer N. Lennon J. T. (2011). The generation and maintenance of diversity in microbial communities. Am. J. Botany 98, 439–448. 10.3732/ajb.1000498 21613137

[B19] Fusco V. Quero G. M. Cho G. S. Kabisch J. Meske D. Neve H.. (2015). The genus weissella: taxonomy, ecology and biotechnological potential. Front. Microbiol. 6, 155. 10.3389/fmicb.2015.00155 25852652 PMC4362408

[B20] Gu Y. Wang Y. Wang P. Wang C. Li M. (2020). Study on the diversity of fungal and bacterial communities in continuous cropping fields of chinese chives (allium tuberosum). BioMed Res. Int. 2020, 1–14. 10.1155/2020/3589758 33381549 PMC7762660

[B21] Han Q. Ma Q. Chen Y. Tian B. Xu L. Bai Y.. (2020). Variation in rhizosphere microbial communities and its association with the symbiotic efficiency of rhizobia in soybean. ISME J. 14, 1915–1928. 10.1038/s41396-020-0648-9 32336748 PMC7367843

[B22] Itumeleng M. Julien T. Tienne Y. (2020). Temporal and spatial interactions modulate the soybean microbiome. FEMS Microbiol. Ecol. 97, 1. 10.1093/femsec/fiaa206 33367840

[B23] J.Y H F.T M L.L S. (2021). Nutrition and aroma components of hotbed chives and leeks in puding county in summer and winter. Food Fermentation Ind. 47, 197–204. 10.13995/j.cnki.11-1802/ts.025522

[B24] Jagannath S. Konappa N. M. Alurappa R. Chowdappa S. (2019). Production, characterization of indole acetic acid and its bioactive potential from endophytic fungi of cymbidium aloifolium l. J. Biol. Active Prod. Nat. 9, 387–409. 10.1080/22311866.2019.1688684

[B25] Katoh K. Misawa K. Kuma K. Miyata T. (2002). Mafft: a novel method for rapid multiple sequence alignment based on fast fourier transform (describes the fft-ns-1, fft-ns-2 and fft-ns-i strategies). Nucl. Acids Res. 30, 3059–3066. 10.1093/nar/gkf436 12136088 PMC135756

[B26] Khan M. S. Gao J. Chen X. Zhang M. Yang F. Du Y.. (2020). Isolation and characterization of plant growth-promoting endophytic bacteria paenibacillus polymyxa sk1 from lilium lancifolium. BioMed Res. Int., 2020:8650957. 10.1155/2020/8650957 32190683 PMC7064867

[B27] Kimura T. Inahashi Y. Matsuo H. Suga T. Iwatsuki M. Shiomi K.. (2018). Pyrizomicin a and b: structure and bioactivity of new thiazolyl pyridines from lechevalieria aerocolonigenes k10-0216. J. Antibiot. Int. J. 71, 606–608. 10.1038/s41429-018-0038-y 29515230

[B28] Kirchman D. L. (2002). The ecology of cytophaga–flavobacteria in aquatic environments. Fems Microbiol. Ecol. 39, 91–100. 10.1016/S0168-6496(01)00206-9 19709188

[B29] Kusstatscher P. Cernava T. Harms K. Maier J. Eigner H. Berg G.. (2019). Disease incidence in sugar beet fields is correlated with microbial diversity and distinct biological markers. Phytobiomes. 3, 22–30. 10.1094/PBIOMES-01-19-0008-R30812563

[B30] Lang C. (2018). Effects of plant endophytic bacteria extracts on growth and yield of corn. Jiangsu Agricul. Sci. 46, 70–73. 10.15889/j.issn.1002-1302.2018.20.015

[B31] Letunic I Bork P. (2007). Interactive tree of life (itol): an online tool for phylogenetic tree display and annotation. Bioinformatics 23, 127–128. 10.1093/bioinformatics/btl529 17050570

[B32] Li W. M. Qin J. Jiao J. G. Liu Q. Y. Huang Z. Y. Wu X. D. (2020). Effects of intercropping garlic combined with nongcanjing on control effect of soft rot, quality and yield of chinese cabbage. J. Changjiang Vegetables 14, 71–74.

[B33] Lian Q. G. Gan L. Qing M. A. Lan X. J. Zhang J. Zong Z. F.. (2016). The disease prevention and growth-promoting effect and mechanism of streptomyces spectabilis sc11 to botrytis cinerea stress. Acta Phytopathologica Sinica 3, 8. 10.13926/j.cnki.apps.2016.03.014

[B34] Liu H. Carvalhais L. C. Crawford M. Singh E. Dennis P. G. Pieterse C. M.. (2017). Inner plant values: diversity, colonization and benefits from endophytic bacteria. Front. Microbiol. 8, 2552. 10.3389/fmicb.2017.02552 29312235 PMC5742157

[B35] Liu L. Zhang L. Cai Z. (2007). A multiple hypothesis test and its application in econometrics. Stat. Res. 4, 26–30. 10.3969/j.issn.1002-4565.2007.04.00731104739

[B36] Lozupone C. Knight R. (2005). Unifrac: a new phylogenetic method for comparing microbial communities. Appl. Environ. Microbiol. 71, 8228–8235. 10.1128/AEM.71.12.8228-8235.2005 16332807 PMC1317376

[B37] Lugtenberg B. Kamilova F. (2009). Plant-growth-promoting rhizobacteria. Ann. Rev. Microbiol. 2009, 541–556. 10.1146/annurev.micro.62.081307.162918 19575558

[B38] Melotto M. Underwood W. He S. Y. (2008). Role of stomata in plant innate immunity and foliar bacterial diseases. Annu. Rev. Phytopathol. 46, 101–122. 10.1146/annurev.phyto.121107.104959 18422426 PMC2613263

[B39] Moin S. Ali S. A. Hasan K. A. Tariq A. Sultana V. Ara J.. (2020). Managing the root rot disease of sunflower with endophytic fluorescent pseudomonas associated with healthy plants. Crop Protect. 130:105066. 10.1016/j.cropro.2019.105066

[B40] Nakashima T. Kamiya Y. Iwatsuki M. Takahashi Y. Omura S. (2014). Mangromicins, six new anti-oxidative agents isolated from a culture broth of the actinomycete, lechevalieria aerocolonigenes k10-0216. J. Antibiot. 67, 533–539. 10.1038/ja.2014.34 24690908

[B41] Ndivo F. M. Njeru E. M. Birgen J. (2018). Efficacy of neem, garlic and aloe extracts in the management of postharvest potato soft rot caused by erwinia carotovora. Asian J. Res. Crop Sci. 1, 1–7. 10.9734/AJRCS/2018/40255

[B42] Ping L. W. Q. J. J. Q. Z. X. L. R. D. Z. Z. J. (2020). Effects of intercropping garlic combined with nongcanjing on control effect of soft rot, quality and yield of chinese cabbage. J. Changjiang Vegetables 14, 71–74. 10.3865/j.issn.1001-3547.2020.14.023

[B43] Price M. N. Dehal P. S. Arkin A. P. (2009). Fasttree: computing large minimum evolution trees with profiles instead of a distance matrix. Mol. Biol. Evol. 26, 1641–1650. 10.1093/molbev/msp077 19377059 PMC2693737

[B44] Reinhold Hurek B. Bünger W. Burbano C. S. Sabale M. Hurek T. (2015). Roots shaping their microbiome: global hotspots for microbial activity. Ann. Rev. Phytopathol. 53, 403–424. 10.1146/annurev-phyto-082712-102342 26243728

[B45] Sauer K. Cooke M. P. (2010). Regulation of immune cell development through soluble inositol-1,3,4,5-tetrakisphosphate. Nat. Rev. Immunol. 10, 257–271. 10.1038/nri2745 20336153 PMC2922113

[B46] Shehata H. R. Ragupathy S. Henry T. A. Newmaster S. G. (2021). Niche specificity and functional diversity of the bacterial communities associated with ginkgo biloba and panax quinquefolius. Sci. Rep. 11:10803. 10.1038/s41598-021-90309-0 34031502 PMC8144622

[B47] Simeon A. U. Abubakar A. (2014). Evaluation of some plant extracts for the control of bacterial soft rot of tubers. J. Exp. Agricul. Int. 4, 1869–1876. 10.9734/AJEA/2014/12309

[B48] Stouvenakers G. Massart S. Depireux P. Hassam M. (2020). Microbial origin of aquaponic water suppressiveness against pythium aphanidermatum lettuce root rot disease. Microorganisms 8, 24. 10.3390/microorganisms8111683 33138322 PMC7694120

[B49] Sun J. Yin G. Y. Ding M. M. Tao Z. X. (2014a). Study on extraction and antioxidant activity of protein from chinese chive seed. Sci. Technol. Food Ind. 35, 291–294. 10.13386/j.issn1002-0306.2014.12.055

[B50] Sun L. Xie Y. Zhang Y. (2014b). Effect of temperature variation within the facilities on antioxidant properties grape leaves under early and delayed cultivation. School Agric. 1, 38–43.

[B51] Tan J. Wang C. Xiang B. Han R. Guo Z. (2013). Hydrogen peroxide and nitric oxide mediated cold- and dehydration-induced myo -inositol phosphate synthase that confers multiple resistances to abiotic stresses. Plant Cell Environ. 36, 288–299. 10.1111/j.1365-3040.2012.02573.x 22774933

[B52] Tan L. Zeng W.-A. Xiao Y. Li P. Gu S. Wu S.. (2021). Fungi-bacteria associations in wilt diseased rhizosphere and endosphere by interdomain ecological network analysis. Front. Microbiol. 12:2536. 10.3389/fmicb.2021.722626 34552573 PMC8450586

[B53] Wang C. Masoudi A. Wang M. Yang J. Shen R. Man M.. (2020a). Community structure and diversity of the microbiomes of two microhabitats at the root–soil interface: implications of meta-analysis of the root-zone soil and root endosphere microbial communities in xiong'an new area. Can. J. Microbiol. 66, 605–622. 10.1139/cjm-2020-0061 32526152

[B54] Wang H. P. Qiu Y. Fang Wei L. I. Song J. P. Zhang X. H. Xi Xiang L. I. (2017a). Investigation and nutrition components analysis of wild chives in hezhang county of guizhou province. J. Plant Gen. Resour. 18, 1137–44.

[B55] Wang J. Zeng X. Zhang H. Li Y. Zhao S. (2018). Effect of exogenous phosphate on the lability and phytoavailability of arsenic in soils. Chemosphere Environ. Toxicol. Risk Assess. 196, 540–547. 10.1016/j.chemosphere.2017.12.191 29329086

[B56] Wang R. Zhang H. Sun L. Qi G. Chen S. Zhao X. (2017b). Microbial community composition is related to soil biological and chemical properties and bacterial wilt outbreak. Sci. Rep. 7, 343. 10.1038/s41598-017-00472-6 28336973 PMC5428506

[B57] Wang X. Ye S. Li G. (2014). Effect on the protection of alcoholic liver injury and antihyperlipidemic of extracts from chinese chives(allium tuberosum). J. Chin. Inst. Food Ence Technol. 14, 28–32.

[B58] Wang X. Zhou X. Cai Z. Guo L. Wang A. (2020b). A biocontrol strain of pseudomonas aeruginosa cq-40 promote growth and control botrytis cinerea in tomato. Pathogens 10, 22. 10.3390/pathogens10010022 33396336 PMC7824093

[B59] Wang Y. Wang C. Gu Y. Wang P. Yang X. (2020c). The variability of bacterial communities in both the endosphere and ectosphere of different niches in chinese chives (allium tuberosum). PLoS ONE 15:e0227671. 10.1371/journal.pone.0227671 31945106 PMC6964972

[B60] Wang Y. Wang C. Wang L. Liu Q. Dong T. Hou P.. (2019). Identification, optimization of fermentation conditions of antagonistic bacterium pseudomonas extremorientalis against botrytis cinerea on pear and evaluation of its biocontrol efficacy. Chin. J. Biol. Control. 35, 437–448. 10.16409/j.cnki.2095-039x.2019.03.007

[B61] Wei G. Ning K. Zhang G. Yu H. Yang S. Dai F.. (2021). Compartment niche shapes the assembly and network of cannabis sativaassociated microbiome. Front. Microbiol. 12:714993. 10.3389/fmicb.2021.714993 34675893 PMC8524047

[B62] Wu X. Shi J. Fang W. X. Guo X. Y. Zhang L. Y. Liu Y. P.. (2019). Allium vegetables are associated with reduced risk of colorectal cancer: a hospital-based matched case-control study in china. Asia Pac. J. Clin. Oncol. 15, e132–e141. 10.1111/ajco.13133 30790463

[B63] Xia F. Wang J. G. Zhu T. Zou B. Rhee S. K. Quan Z. X. (2018). Ubiquity and diversity of complete ammonia oxidizers (comammox). Appl. Environ. Microbiol. 84:e01390–18. 10.1128/AEM.01390-18 30315079 PMC6275355

[B64] Xie J. Wang X. Xu J. Xie H. Cai Y. Liu Y.. (2021). Strategies and structure feature of the aboveground and belowground microbial community respond to drought in wild rice (oryza longistaminata). Rice 14, 1–17. 10.1186/s12284-021-00522-8 34495440 PMC8426455

[B65] Xu L. Yang F. Wu X. Ding R. Lu Y. Hou B. (2016). Effect of intercropping with onion or garlic on yield and quality of chinese cabbage. Northern Horticul. 14, 6–10. 10.11937/bfyy.201614002

[B66] Yang Z. Cao J. (2016). Research progress of endophytic fungi and their secondary metabolites. J. Microbiol. 36, 1–6. 10.3969/j.issn.1005-7021.2016.04.001

[B67] Zhang B. Zhang J. Liu Y. Shi P. Wei G. (2018). Co-occurrence patterns of soybean rhizosphere microbiome at a continental scale. Soil Biol. Biochem. 118, 178–186. 10.1016/j.soilbio.2017.12.011

[B68] Zhu B. Wu J. Ji Q. Wu W. Qin L. (2020). Diversity of rhizosphere and endophytic fungi in atractylodes macrocephala during continuous cropping. PeerJ 8:e8905. 10.7717/peerj.8905 32292655 PMC7144587

[B69] Zhu L. Qian N. Sun Y. Lu X. Qian L. (2021). Pseudomonas fluorescens dn16 enhances cucumber defense responses against the necrotrophic pathogen botrytis cinerea by regulating thermospermine catabolism. Front. Plant Sci. 12:645338. 10.3389/fpls.2021.645338 33692821 PMC7937916

[B70] Zhuang L. Li Y. Wang Z. Yu Y. Zhang N. Yang C.. (2020). Synthetic community with six pseudomonas strains screened from garlic rhizosphere microbiome promotes plant growth. Microb. Biotechnol. 14, 488–502. 10.1111/1751-7915.13640 32762153 PMC7936309

[B71] ZiLi M. Mei-jin Y. Yan-Mei Y. Wei Z. Xiang-Quan Y. Qian Z.. (2018). Soil microorganism quantity and soil enzyme activity in the wheat-garlic intercropping system. J. Agricul. Resour. Environ. 35, 430–438. 10.13254/j.jare.2018.0080

