# Supplementary material for: Colonization and Interaction of Bacteria Associated With Chinese Chives Affected by Ecological Compartments and Growth Conditions
Source: Front Microbiol. 2022 Feb 14;13:775002. doi: 10.3389/fmicb.2022.775002 (PMC8883035; doi:10.3389/fmicb.2022.775002)
Supplement: Supplementary file 1 [file Data_Sheet_1.DOCX]

 STUDY: PRJNA742175
  SAMPLE: HYg_J2 (SAMN19931367)
    EXPERIMENT: HYg_J2 (SRX12201814)
      RUN: raw.split.HYg_J2.1.fq (SRR15911408)
  SAMPLE: HYg_J1 (SAMN19931366)
    EXPERIMENT: HYg_J1 (SRX12201813)
      RUN: raw.split.HYg_J1.1.fq (SRR15911409)
  SAMPLE: HYg_J3 (SAMN19931368)
    EXPERIMENT: HYg_J3 (SRX12201815)
      RUN: raw.split.HYg_J3.1.fq (SRR15911407)
  SAMPLE: HYg_L1 (SAMN19931369)
    EXPERIMENT: HYg_L1 (SRX12201816)
      RUN: raw.split.HYg_L1.1.fq (SRR15911406)
  SAMPLE: HYL_L1 (SAMN19931339)
    EXPERIMENT: HYL_L1 (SRX12201812)
      RUN: raw.split.HYL_L1.1.fq (SRR15911410)
  SAMPLE: HYP_S3 (SAMN19931365)
    EXPERIMENT: HYP_S3 (SRX12201811)
      RUN: raw.split.HYP_S3.1.fq (SRR15911411)
  SAMPLE: HYP_S2 (SAMN19931364)
    EXPERIMENT: HYP_S2 (SRX12201810)
      RUN: raw.split.HYP_S2.1.fq (SRR15911412)
  SAMPLE: HYP_S1 (SAMN19931363)
    EXPERIMENT: HYP_S1 (SRX12201809)
      RUN: raw.split.HYP_S1.1.fq (SRR15911413)
  SAMPLE: HYP_R3 (SAMN19931362)
    EXPERIMENT: HYP_R3 (SRX12201808)
      RUN: raw.split.HYP_R3.1.fq (SRR15911414)
  SAMPLE: HYP_R2 (SAMN19931361)
    EXPERIMENT: HYP_R2 (SRX12201807)
      RUN: raw.split.HYP_R2.1.fq (SRR15911415)
  SAMPLE: HYP_R1 (SAMN19931360)
    EXPERIMENT: HYP_R1 (SRX12201806)
      RUN: raw.split.HYP_R1.1.fq (SRR15911416)
  SAMPLE: HYP_M3 (SAMN19931359)
    EXPERIMENT: HYP_M3 (SRX12201805)
      RUN: raw.split.HYP_M3.1.fq (SRR15911417)
  SAMPLE: HYP_M1 (SAMN19931357)
    EXPERIMENT: HYP_M1 (SRX12201803)
      RUN: raw.split.HYP_M1.1.fq (SRR15911419)
  SAMPLE: HYP_M2 (SAMN19931358)
    EXPERIMENT: HYP_M2 (SRX12201804)
      RUN: raw.split.HYP_M2.1.fq (SRR15911418)
  SAMPLE: HYP_L3 (SAMN19931356)
    EXPERIMENT: HYP_L3 (SRX12201802)
      RUN: raw.split.HYP_L3.1.fq (SRR15911420)
  SAMPLE: HYP_L1 (SAMN19931354)
    EXPERIMENT: HYP_L1 (SRX12201799)
      RUN: raw.split.HYP_L1.1.fq (SRR15911423)
  SAMPLE: HYL_J3 (SAMN19931338)
    EXPERIMENT: HYL_J3 (SRX12201801)
      RUN: raw.split.HYL_J3.1.fq (SRR15911421)
  SAMPLE: HYP_L2 (SAMN19931355)
    EXPERIMENT: HYP_L2 (SRX12201800)
      RUN: raw.split.HYP_L2.1.fq (SRR15911422)
  SAMPLE: HYP_J3 (SAMN19931353)
    EXPERIMENT: HYP_J3 (SRX12201798)
      RUN: raw.split.HYP_J3.1.fq (SRR15911424)
  SAMPLE: LB_L1 (SAMN19931384)
    EXPERIMENT: LB_L1 (SRX12201832)
      RUN: raw.split.LB_L1.1.fq (SRR15911390)
  SAMPLE: HYP_J2 (SAMN19931352)
    EXPERIMENT: HYP_J2 (SRX12201797)
      RUN: raw.split.HYP_J2.1.fq (SRR15911425)
  SAMPLE: HYP_J1 (SAMN19931351)
    EXPERIMENT: HYP_J1 (SRX12201796)
      RUN: raw.split.HYP_J1.1.fq (SRR15911426)
  SAMPLE: HYL_S3 (SAMN19931350)
    EXPERIMENT: HYL_S3 (SRX12201795)
      RUN: raw.split.HYL_S3.1.fq (SRR15911427)
  SAMPLE: HYL_S2 (SAMN19931349)
    EXPERIMENT: HYL_S2 (SRX12201794)
      RUN: raw.split.HYL_S2.1.fq (SRR15911428)
  SAMPLE: HYL_S1 (SAMN19931348)
    EXPERIMENT: HYL_S1 (SRX12201793)
      RUN: raw.split.HYL_S1.1.fq (SRR15911429)
  SAMPLE: HYL_R3 (SAMN19931347)
    EXPERIMENT: HYL_R3 (SRX12201792)
      RUN: raw.split.HYL_R3.1.fq (SRR15911430)
  SAMPLE: HYL_R2 (SAMN19931346)
    EXPERIMENT: HYL_R2 (SRX12201791)
      RUN: raw.split.HYL_R2.1.fq (SRR15911431)
  SAMPLE: LB_M3 (SAMN19931389)
    EXPERIMENT: LB_M3 (SRX12201838)
      RUN: raw.split.LB_M3.1.fq (SRR15911384)
  SAMPLE: LB_R3 (SAMN19931392)
    EXPERIMENT: LB_R3 (SRX12201841)
      RUN: raw.split.LB_R3.1.fq (SRR15911381)
  SAMPLE: HYL_J2 (SAMN19931337)
    EXPERIMENT: HYL_J2 (SRX12201790)
      RUN: raw.split.HYL_J2.1.fq (SRR15911432)
  SAMPLE: HYL_J1 (SAMN19931336)
    EXPERIMENT: HYL_J1 (SRX12201789)
      RUN: raw.split.HYL_J1.1.fq (SRR15911433)
  SAMPLE: HYg_L2 (SAMN19931370)
    EXPERIMENT: HYg_L2 (SRX12201817)
      RUN: raw.split.HYg_L2.1.fq (SRR15911405)
  SAMPLE: HYg_L3 (SAMN19931371)
    EXPERIMENT: HYg_L3 (SRX12201818)
      RUN: raw.split.HYg_L3.1.fq (SRR15911404)
  SAMPLE: HYg_M1 (SAMN19931372)
    EXPERIMENT: HYg_M1 (SRX12201819)
      RUN: raw.split.HYg_M1.1.fq (SRR15911403)
  SAMPLE: HYg_M2 (SAMN19931373)
    EXPERIMENT: HYg_M2 (SRX12201820)
      RUN: raw.split.HYg_M2.1.fq (SRR15911402)
  SAMPLE: HYg_M3 (SAMN19931374)
    EXPERIMENT: HYg_M3 (SRX12201821)
      RUN: raw.split.HYg_M3.1.fq (SRR15911401)
  SAMPLE: HYg_R1 (SAMN19931375)
    EXPERIMENT: HYg_R1 (SRX12201822)
      RUN: raw.split.HYg_R1.1.fq (SRR15911400)
  SAMPLE: HYg_R2 (SAMN19931376)
    EXPERIMENT: HYg_R2 (SRX12201824)
      RUN: raw.split.HYg_R2.1.fq (SRR15911398)
  SAMPLE: HYg_R3 (SAMN19931377)
    EXPERIMENT: HYg_R3 (SRX12201825)
      RUN: raw.split.HYg_R3.1.fq (SRR15911397)
  SAMPLE: HYg_S1 (SAMN19931378)
    EXPERIMENT: HYg_S1 (SRX12201826)
      RUN: raw.split.HYg_S1.1.fq (SRR15911396)
  SAMPLE: LB_J2 (SAMN19931382)
    EXPERIMENT: LB_J2 (SRX12201830)
      RUN: raw.split.LB_J2.1.fq (SRR15911392)
  SAMPLE: LB_J3 (SAMN19931383)
    EXPERIMENT: LB_J3 (SRX12201831)
      RUN: raw.split.LB_J3.1.fq (SRR15911391)
  SAMPLE: LB_L2 (SAMN19931385)
    EXPERIMENT: LB_L2 (SRX12201833)
      RUN: raw.split.LB_L2.1.fq (SRR15911389)
  SAMPLE: LB_L3 (SAMN19931386)
    EXPERIMENT: LB_L3 (SRX12201835)
      RUN: raw.split.LB_L3.1.fq (SRR15911387)
  SAMPLE: LB_M1 (SAMN19931387)
    EXPERIMENT: LB_M1 (SRX12201836)
      RUN: raw.split.LB_M1.1.fq (SRR15911386)
  SAMPLE: LB_M2 (SAMN19931388)
    EXPERIMENT: LB_M2 (SRX12201837)
      RUN: raw.split.LB_M2.1.fq (SRR15911385)
  SAMPLE: LB_R2 (SAMN19931391)
    EXPERIMENT: LB_R2 (SRX12201840)
      RUN: raw.split.LB_R2.1.fq (SRR15911382)
  SAMPLE: LB_R1 (SAMN19931390)
    EXPERIMENT: LB_R1 (SRX12201839)
      RUN: raw.split.LB_R1.1.fq (SRR15911383)
  SAMPLE: HYL_M1 (SAMN19931342)
    EXPERIMENT: HYL_M1 (SRX12201845)
      RUN: raw.split.HYL_M1.1.fq (SRR15911377)
  SAMPLE: HYL_L2 (SAMN19931340)
    EXPERIMENT: HYL_L2 (SRX12201823)
      RUN: raw.split.HYL_L2.1.fq (SRR15911399)
  SAMPLE: HYg_S2 (SAMN19931379)
    EXPERIMENT: HYg_S2 (SRX12201827)
      RUN: raw.split.HYg_S2.1.fq (SRR15911395)
  SAMPLE: HYg_S3 (SAMN19931380)
    EXPERIMENT: HYg_S3 (SRX12201828)
      RUN: raw.split.HYg_S3.1.fq (SRR15911394)
  SAMPLE: LB_J1 (SAMN19931381)
    EXPERIMENT: LB_J1 (SRX12201829)
      RUN: raw.split.LB_J1.1.fq (SRR15911393)
  SAMPLE: HYL_L3 (SAMN19931341)
    EXPERIMENT: HYL_L3 (SRX12201834)
      RUN: raw.split.HYL_L3.1.fq (SRR15911388)
  SAMPLE: LB_W1 (SAMN19931393)
    EXPERIMENT: LB_W1 (SRX12201842)
      RUN: raw.split.LB_W1.1.fq (SRR15911380)
  SAMPLE: LB_W2 (SAMN19931394)
    EXPERIMENT: LB_W2 (SRX12201843)
      RUN: raw.split.LB_W2.1.fq (SRR15911379)
  SAMPLE: LB_W3 (SAMN19931395)
    EXPERIMENT: LB_W3 (SRX12201844)
      RUN: raw.split.LB_W3.1.fq (SRR15911378)
  SAMPLE: HYL_M2 (SAMN19931343)
    EXPERIMENT: HYL_M2 (SRX12201846)
      RUN: raw.split.HYL_M2.1.fq (SRR15911376)
  SAMPLE: HYL_M3 (SAMN19931344)
    EXPERIMENT: HYL_M3 (SRX12201847)
      RUN: raw.split.HYL_M3.1.fq (SRR15911375)
  SAMPLE: HYL_R1 (SAMN19931345)
    EXPERIMENT: HYL_R1 (SRX12201848)
      RUN: raw.split.HYL_R1.1.fq (SRR15911374)
