# Supplementary material for: Colonization and Interaction of Bacteria Associated With Chinese Chives Affected by Ecological Compartments and Growth Conditions
Source: Front Microbiol. 2022 Feb 14;13:775002. doi: 10.3389/fmicb.2022.775002 (PMC8883035; doi:10.3389/fmicb.2022.775002)
Supplement: Supplementary file 2 [file Data_Sheet_2.PDF]

Supplementary Material

1 TABLE

Table S1. Soil physical and chemical properties in different growth conditions

	Solar greenhouse	Arched shed	Open field
Air temperature(°C)	24.4	18	17
Relative humidity (%)	96	46.7	37.5
Soil temperature (°C)	19.50±0.87a	17.43±0.51ab	13.80±0.20b
Soil moisture content (%)	13.05±0.11a	12.10±0.19ab	11.39±0.28b
Power of hydrogen (pH)	6.12±0.08a	6.55±0.11a	6.11±0.26a
Total organic carbon (g/kg)	11.35±2.17a	8.73±0.53ab	5.64±0.66b
Total nitrogen (g/kg)	1.72±0.04a	1.28±0.07a	1.66±0.06a
Total phosphorus (g/kg)	3.14±0.21ab	1.89±0.04b	3.57±0.12a
Total potassium (g/kg)	8.67±1.05a	5.49±0.17a	9.95±0.58a
Ammonium nitrogen (mg/kg)	85.74±1.32ab	55.07±2.74b	91.66±2.93a
Available phosphorus (mg/kg)	161.81±2.51a	142.64±0.51ab	130.31±3.61b
Rapidly available potassium (mg/kg)	366.83±8.06ab	218.47±11.65b	468.31±6.46a

2 FIGURES

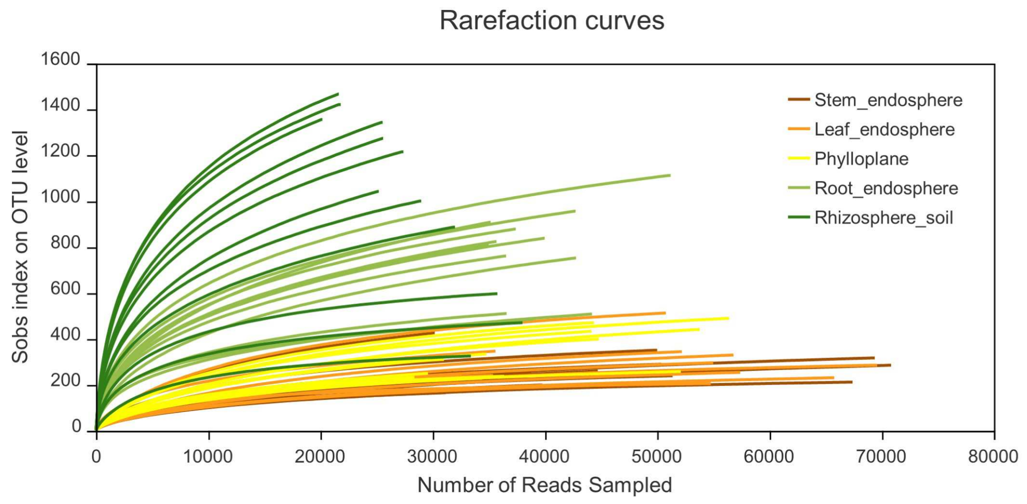

Figure S1. The rarefaction curves when grouped by ecological compartments.

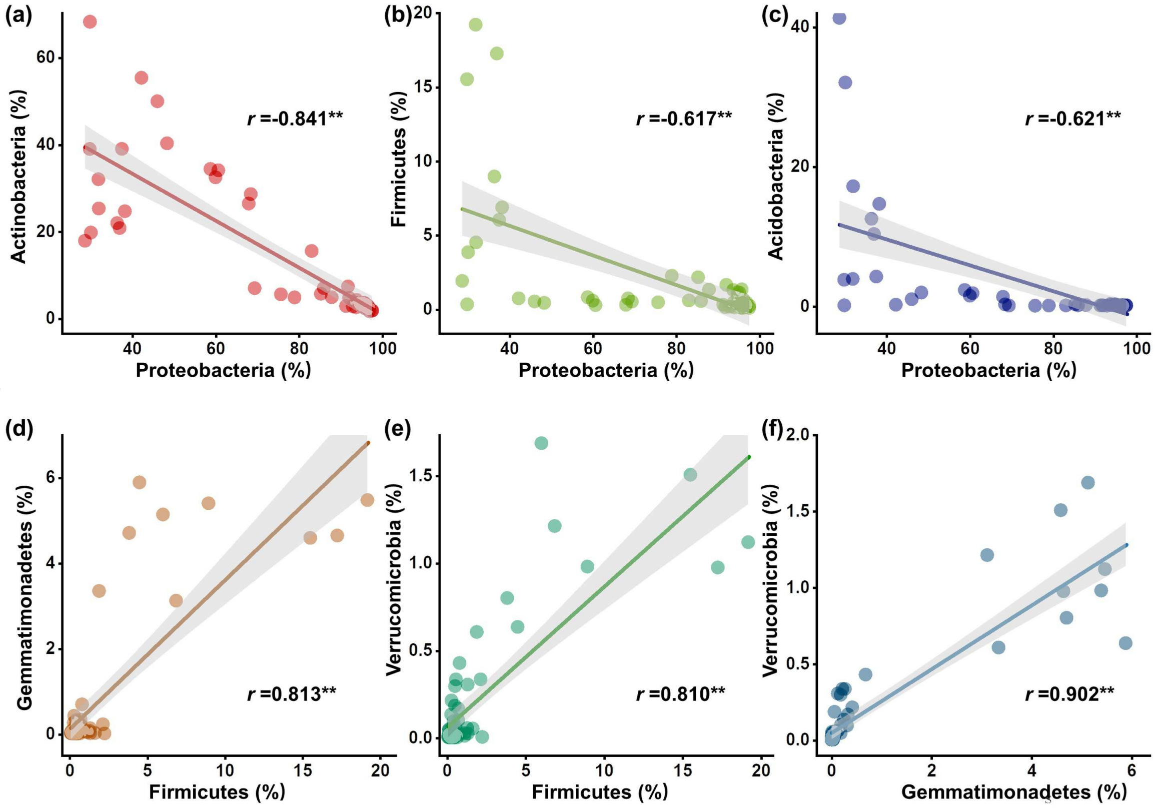

Figure S2. Analysis of all samples at phylum level using pearson's correlation coef-ficient, Proteobacteria and Actinobacteria. **(a)**, Proteobacteria and Firmicutes **(b)**, Proteobacteria and Acidobacteria **(c)**, Firmicutes and Gemmatimonadetes **(d)**, Firmicutes and Verrucomicrobia **(e)**, Gemmatimonadetes and Verrucomicrobia **(f)**. Points represent the relative abundance of two phyla in each sample, and the grey region is Confidence band. $*P \leq 0.05$. $**P \leq 0.01$. $***P \leq 0.001$.

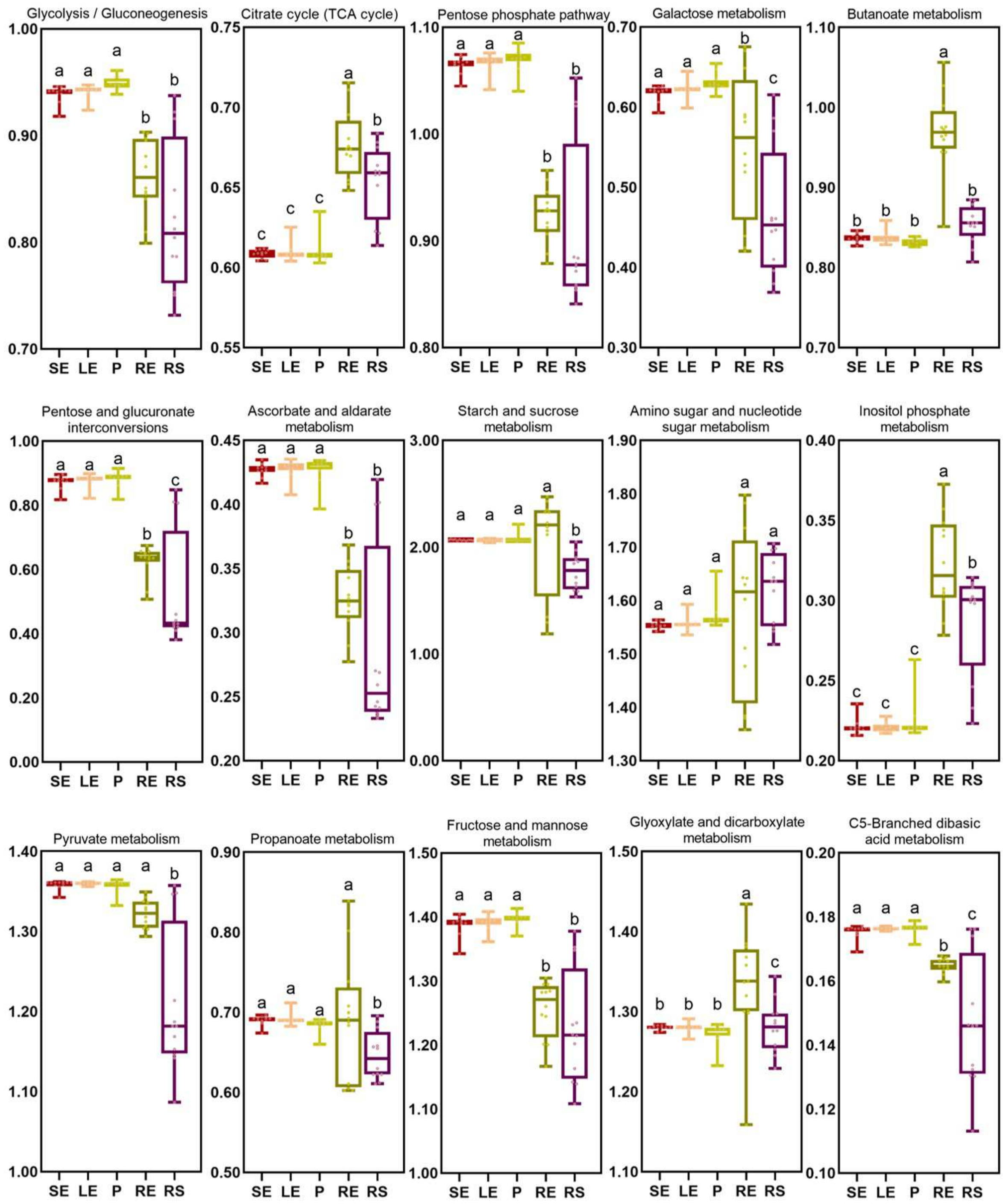

Figure S3. The inter group difference test of the top 15 tertiary metabolic pathways with the highest abundance in the secondary metabolic pathway Carbohydrate metabolism. The ordinate represents the abundance value of each pathway, SE, stem endosphere; LE, leaf endosphere; P, phylloplane ; RE, root endosphere; R, rhizosphere. The horizontal bars within boxes represent medians. The tops and bottoms of boxes represent the 75th and 25th percentiles, respectively. The aboveground and lower whiskers extend to data no more than 1.5× the interquartile range from the aboveground edge and lower edge of the box, respectively. Different letters indicate display differences between groups ($P < 0.05$, ANOVA, Tukey-HSD test). The numbers of replicated samples in this figure are as follows: in different ecological compartments, n = 12. (Supplementary Materials).
